# Integrative model of leukocyte genomics and organ dysfunction in heart failure patients requiring mechanical circulatory support: a prospective observational study

**DOI:** 10.1186/s12920-017-0288-8

**Published:** 2017-08-29

**Authors:** Nicholas Wisniewski, Galyna Bondar, Christoph Rau, Jay Chittoor, Eleanor Chang, Azadeh Esmaeili, Martin Cadeiras, Mario Deng

**Affiliations:** 1 Department of Medicine, Division of Cardiology, University of California Los Angeles, 100 UCLA Medical Plaza, Suite 630, Los Angeles, California 90095 USA; 2 Department of Integrative Biology and Physiology, University of California Los Angeles, 612 Charles E. Young Drive East, Los Angeles, California 90095 USA; 3 Department of Anesthesiology, Division of Molecular Medicine, University of California Los Angeles, 100 UCLA Medical Plaza, Suite 630, Los Angeles, California 90095 USA

**Keywords:** Integrative model, Leukocyte genomics, Organ dysfunction, Heart failure, Mechanical circulatory support

## Abstract

**Background:**

The implantation of mechanical circulatory support devices in heart failure patients is associated with a systemic inflammatory response, potentially leading to death from multiple organ dysfunction syndrome. Previous studies point to the involvement of many mechanisms, but an integrative hypothesis does not yet exist. Using time-dependent whole-genome mRNA expression in circulating leukocytes, we constructed a systems-model to improve mechanistic understanding and prediction of adverse outcomes.

**Methods:**

We sampled peripheral blood mononuclear cells from 22 consecutive patients undergoing mechanical circulatory support device (MCS) surgery, at 5 timepoints: day −1 preoperative, and postoperative days 1, 3, 5, and 8. Clinical phenotyping was performed using 12 clinical parameters, 2 organ dysfunction scoring systems, and survival outcomes. We constructed a strictly phenotype-driven time-dependent non-supervised systems-representation using weighted gene co-expression network analysis, and annotated eigengenes using gene ontology, pathway, and transcription factor binding site enrichment analyses. Genes and eigengenes were mapped to the clinical phenotype using a linear mixed-effect model, with Cox models also fit at each timepoint to survival outcomes.

**Results:**

We inferred a 19-module network, in which most module eigengenes correlated with at least one aspect of the clinical phenotype. We observed a response of advanced heart failure patients to surgery orchestrated into stages: first, activation of the innate immune response, followed by anti-inflammation, and finally reparative processes such as mitosis, coagulation, and apoptosis. Eigengenes related to red blood cell production and extracellular matrix degradation became predictors of survival late in the timecourse corresponding to multiorgan dysfunction and disseminated intravascular coagulation.

**Conclusions:**

Our model provides an integrative representation of leukocyte biology during the systemic inflammatory response following MCS device implantation. It demonstrates consistency with previous hypotheses, identifying a number of known mechanisms. At the same time, it suggests novel hypotheses about time-specific targets.

**Electronic supplementary material:**

The online version of this article (doi:10.1186/s12920-017-0288-8) contains supplementary material, which is available to authorized users.

## Background

Mechanical circulatory support (MCS) device therapy is a treatment option for patients with advanced heart failure (AdHF). It consists of surgical implantation of a mechanical device to assist or replace cardiac function and restore normal hemodynamics. It is commonly used as a bridge to heart transplantation for patients deteriorating while awaiting an organ transplant, as a bridge to recovery when myocardial function can still be restored, and as lifelong (destination) therapy for patients who are not candidates for transplantation [1]. The physiological rationale underlying the treatment is that by restoring normal hemodynamics, it is possible to at least partially restore systemic functions such as oxygen metabolism and neurohormonal regulation, which in turn reduces hypoxia and inflammation, and improves the function of all organs promoting the recovery of the heart failure patient [2].

**Fig. 1:**
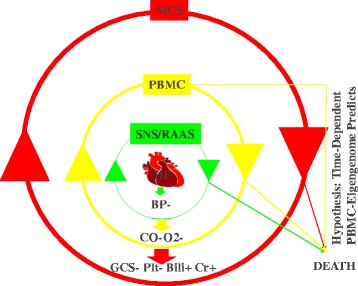
Neuro-Endocrino-Immuno-Pathophysiology Of Heart Failure. **HF**: Heart failure (HF) is initiated by various mechanisms. HF leads to lower mean arterial pressure (**BP-**) and compensatory upregulation of sympathetic nervous system (**SNS**) and renin-angiotensin-aldosterone system (**RAAS**) in an attempt to maintain BP, cardiac output (CO) and oxygen delivery (O2). Further myocardial injury leads to HF progression and reduced CO (**CO-**) and O2–delivery (**O2-**) to organs/tissues. This triggers, hypothetically via organ-specific endothelial cell/platelet/peripheral blood mononuclear cell (**PBMC**) interactions, compensatory immune system activation that serves to provide shortterm compensation for the failing heart. This increasing immune system activation coincides with worsening organ dysfunction of the kidneys (higher creatinine, **Cr+**), liver (higher bilirubin, **Bili+**), bone marrow (lower platelets, **Plt-**) and brain (worse Glasgow Coma Scale, **GCS-**). In this milieu, the surgical implantation of therapies such as mechanical circulatory support (**MCS**) restore normal CO and O2–delivery to the organs yet are associated with an unpredictable inflammatory state transition. This makes the prediction of a future survivor/non-survivor outcome challenging. We hypothesize that time-dependent NGS-based PBMC-biology analysis, when added to current clinical predictors, improve prediction precision of future survivor/non-survivor outcome

However, up to 20% of AdHF-patients die during the first year post MCS-surgery. Surgical implantation of the device is a major procedure, associated with a systemic inflammatory response, which most often is self-limited but lack of self-control can potentially lead to an uncontrollable inflammatory response, acute organ dysfunction, and ultimately to patient’s death. Clinically, patients develop a cascade of complications, including hepatic, renal, pulmonary, immunologic, hematologic, gastro-intestinal, metabolic and neurologic dysfunction. It is presumed that an altered immune response induced by both the MCS device and surgery, facilitated by the preexisting HF syndrome [3] and its sequentially escalating stages of compensation first described more than 50 years ago [4], is responsible. The leading cause of mortality in intensive care units and the most feared consequence of this systemic inflammatory response is the Multiple Organ Dysfunction Syndrome (MOD) [5]. Heart failure (HF), initiated by various mechanisms, leads to compensatory chronic upregulation of sympathetic nervous system (SNS) and renin-angiotensin-aldosterone system (RAAS) activity in an attempt to maintain BP, cardiac output (CO) and oxygen delivery (O2). Further progression of myocardial injury and HF progression leads to reduced CO and O2–delivery to organs/tissues. This triggers, hypothetically via organ-specific endothelial cell (EC)/platelet (PLT)/peripheral blood mononuclear cell (PBMC) interactions, compensatory immune system activation, first described by measuring elevated tumor-necrosis factor –alpha levels in patients with most severe HF [6], that hypothetically serves to provide short-term compensation for the failing heart. This increasing immune system activation coincides with worsening organ dysfunction of the kidneys (higher creatinine, Cr), liver (higher bilirubin, Bili), bone marrow (lower platelets) and brain (worse Glasgow Coma Scale, GCS). In this milieu, therapies such as the implantation of MCS restore normal CO and O2–delivery to the organs yet are associated with an unpredictable inflammatory state transition, and associated risk of organ dysfunction and death [1, 7, 8] (Fig. 1).

It is thought that activation of the immune -associated inflammatory and coagulation pathway produces a fibrin matrix that blocks blood flow in the microcirculation causing tissue necrosis, ultimately leading to organ failure [9]. Few therapeutic options are currently established to help reduce this risk, as most aspects of the progression to MOD are not well understood. For example, anti-inflammatory measures such as glucocorticoids have yielded mixed results [10], and are therefore not routinely used in the management of these patients. Successful therapeutic strategies apparently require the understanding of the multiple systems involved and the factors controlling their emergent properties or effects.

A significant amount of work has been done examining similar therapies for systemic infection, i.e. sepsis–related MOD [11]. In both sepsis- and MCS-related MOD, the interplay between leukocytes, platelets, and endothelium is thought to play a critical role in mediating the phenotype [12, 13], and so many of the strategies are understood as affecting various aspects of the endothelial response to inflammatory injury. For example, a number of clinical trials have been conducted to evaluate therapies targeted at controlling cell adhesion, coagulation cascade and apoptosis such as transcription factors like NF-κB; signaling pathways such as MAPK; or vasoactive drugs to counteract the vascular endothelial response to inflammation such as nitric oxide synthase inhibitors [14]. Unfortunately, most interventions have failed to improve outcomes, leaving much work to be done researching new therapeutic targets but also identifying new approaches to understand the system and its controlling factors to be able to intervene using a systems-based strategy.

*There is currently no hypothesis about the integrative systems biology* in MCS-related MOD*,* a key element to understand the evolution of a phenotype and development of new therapeutic strategies. The coordination of all these features has yet to be contextualized, and many questions remain about how they are orchestrated together as a time-evolving system. Analysis of phenotype-driven longitudinal whole-genome mRNA expression in circulating leukocytes offers a way to synchronously conceptualize all of these known features, while also facilitating discovery of novel elements. Several machine learning and bioinformatics tools are available to assist in statistical modeling of high-dimensional data, and have yielded promising results in many genomic studies. These methods allow for discovery of interesting temporal modifications of the biology of the immune response and recovery process, and identify systemic motifs that are of clinical importance.

In this article, we apply whole-genome mRNA gene expression profiling in circulating peripheral blood mononuclear cells (PBMC). PBMC is commonly used to study leukocyte gene expression because it is easily accessible and includes several key inflammatory cell populations that play a central role in the orchestration of the immune response during health and disease. To reconstruct the temporal dynamics in response to surgery during the critical early hazard period, we analyzed a time-series of samples taken at baseline and during the early and most critical period of the MCS therapy creating a 5 time-point time-course study conducted in the sickest patients suffering of AdHF. Because most of the dynamic response to surgery occurs in the first postoperative week, it is expected that patient trajectories are most sensitive and informative during this window. Our analysis strategy then uses machine learning to gain a comprehensive picture of the systems biology and its relation to time-dependent clinical phenotypes and outcomes.

## Methods

### Patients

We collected blood samples from 22 consecutive AdHF patients admitted to our hospital undergoing MCS between March 2013 and September 2014. Samples were collected at 5 timepoints: day −1 preoperative, and postoperative days 1, 3, 5, and 8. The study was conducted under UCLA IRB 12–000351 approval and all patients signed informed consent to participate. To address to most pressing clinical problem of MCS-related perioperative MOD [1, 7, 8], we chose to base this analysis on AdHF-patients undergoing MCS-surgery alone.

### Clinical management

All study participants were referred to the UCLA Integrated AdHF Program and evaluated for the various therapeutic options, including continued optimal medical management, MCS, and heart transplantation. All study participants were recommended by the multidisciplinary heart transplant selection committee to undergo MCS-surgery therapy, and consented to proceed. Preoperatively, *n* = 2 patients were in a state of critical cardiogenic shock (Interagency Registry for Mechanically Assisted Circulatory Support (INTERMACS) class 1); *n* = 12 patients were progressively declining despite being on inotropic support (INTERMACS class 2); *n* = 7 patients were stable but inotrope dependent (INTERMACS class 3); and *n* = 1 patient had resting failure symptoms (INTERMACS class 4) [1]. All patients were optimized regarding medical heart failure therapy, and underwent MCS-therapy according to established guidelines [15, 16].

After anesthesia induction, patients were intubated and placed on cardiopulmonary bypass. The type of MCS-device selected depended on the acuity and severity of the heart failure syndrome as well as patient characteristics [17]. For left ventricular support, patients underwent either Heartmate II (HeartMate II® pumps are valveless, rotary, continuous flow pumps) or HVAD (HeartWare® HVAD pumps are valveless, centrifugal, continuous flow pumps). For biventricular support, patients underwent either Centrimag-BVAD (Centrimag® pumps are valveless, centrifugal, continuous flow pumps that are external to the body), PVAD biventricular assist device (BVAD) (Thoratec® Paracorporeal Ventricular Assist Device (PVAD) pumps each contain two mechanical tilting disk valves) or the t-TAH (the Temporary Total Artificial Heart consists of two artificial ventricles that are used to replace the failing heart).

Various combinations of cardiovascular inotropic and vasoactive drugs were used to support patient’s hemodynamics postoperatively, tailored to individualized requirements. In addition, other temporary organ system support was administered as required (e.g. mechanical ventilation, hemodialysis, blood transfusions, antibiotic therapy).

### Clinical phenotyping

Demographic variables were obtained for all patients, and we also collected 12 distinct parameters on a daily basis for time-dependent clinical phenotyping of the patient cohort. At each timepoint, a comprehensive set of clinical variables to assess multiple organ dysfunction were recovered from patient records including: serum bilirubin, serum creatinine, leukocyte count, platelet count, alveolar oxygen pressure, fraction of inspired oxygen (FiO2), mean systemic arterial pressure (MAP), INR (International Normalized Ratio, for prothrombin time), blood glucose, heart rate, respiratory rate, temperature, and the Glasgow Coma Scale (GCS).

Using combinations of these parameters, we also calculated two validated and commonly used composite organ dysfunction scores, the Sequential Organ Failure Assessment (SOFA) score [18] and Model of Endstage Liver Disease without except International Normalized Ratio (INR) (MELD-XI) [19]. The SOFA score is a validated and widely accepted measure that rates degree of organ failure across 6 major organ systems (cardiovascular, respiratory, neurological, renal, hepatic, and coagulation). The MELD-XI score is a variation of the MELD score that uses only the bilirubin and creatinine levels, and eliminates the INR, which is typically not interpretable in these patients given the need of anticoagulation. Additional information can be found in Additional file 1.

### Sample processing

#### Sample collection and RNA isolation

Phlebotomy was performed at prespecified timepoints including day −1 preoperative, and postoperative days 1, 3, 5, and 8. Mononuclear cells were isolated from 8 ml of blood collected by Vacutainer Cell Preparation Tubes (CPT) with sodium citrate (Becton Dickinson, Franklin Lakes, NJ) and resuspended in RNA Protect (Qiagen, Valencia, CA) within 2 h of phlebotomy. Based on the successful AlloMapTM test development which was the first FDA-cleared PBMC-GEP-based In-Vitro-Diagnostic Multivariate Index Assay (IVDMIA) that maps the interaction of a transplanted heart’s allo-endothelial cells with the recipient’s leukocytes [20, 21] and preliminary data for the current project [22, 23] and its planned clinical utility, we chose the same mixed PBMC population, refraining from isolated subpopulation analyses.

We collected blood in Vacutainer Cell Preparation Tubes (CPT) with sodium citrate (Becton Dickinson, Franklin Lakes, NJ). Peripheral blood mononuclear cells (PBMC) were isolated using manufactural protocol. In short: Samples were processed within 2 h after blood collection. The CPT tube was centrifuged at room temperature (22 °C) for 20 min at 3000RPM or 2000RCF. Plasma was separated without disturbing the cell layer. The cell layer was collected, washed with Phosphate Buffered Solution (PBS)(Thermo Fisher Scientific, Woodlend Hills, CA), and centrifuged again for 20 min at 1135 RMP or 300RCF at 22 °C. The supernatant was aspirated, the pellet was washed with PBS, and centrifuged for 20 min at 5.6 RPM at 4 °C. The supernatant was discarded. The pellet was dissolved in 0.5 ml of RNA protect (Qiagen, Valencia, CA), frozen, and stored at -80 °C. The RNA was isolated from the PBMC using RNeasy Mini Kit (Qiagen, Valencia, CA). The purity and concentration of the RNA was checked by NanoDrop® ND-1000 spectrophotometer (NanoDrop Technologies, Wilmington, DE).. The concentration was 50 ng/ml. The purity was 260/280 ~ 2.0. The integrity of the RNA was analyzed by Agilent® 2100 Bioanalyzer (Agilent Technologies, Palo Alto, CA);. RIN > 9.0 and average > 9.5.

#### RNA processing and analysis

After RNA extraction, quantification and quality assessment, total mRNA was amplified and subjected to NGS transcriptome analysis on the Illumina HiSeq2000 TruSeq. Data was then subjected to quantile normalization using GenomeStudio (Illumina, San Diego, CA). Batch effects were removed using the ‘ComBat’ algorithm in R [24].

Prior to analysis, the entire set of mRNA transcripts (39,740) was filtered by variance and entropy criteria to remove uninformative transcripts. Filtering improves the interpretability of the network analysis, as well as reduces the bias in cluster analysis [25]. We therefore first removed genes with zero variance, and then placed cuts on the remaining transcripts to remove the lower 30th quantiles, reducing our list of transcripts to 14,753.

### Statistical analysis

We used weighted gene co-expression network analysis (WGNCA) [26] to compute clustered co-expressed gene modules and infer an eigengene network. The eigengene approach offers a powerful way to reduce dimensionality while maintaining biological interpretability, effectively de-noising the data for a larger systems analysis. The result provides a useful framework for relational and mechanistic reasoning.

We interpreted the eigengenes by statistically relating them to clinical and biological features. We inferred each module’s clinical relevance using a linear mixed-effect model, connecting the module eigengenes to the panel of day-to-day clinical parameters. We then inferred the biological relevance of each module using bioinformatics tools: gene ontology (GOSim [27]), pathway (Strand NGS [28]), and transcription factor binding site (rVista [29]) enrichment analyses.

We then inferred temporal patterns in both the eigengenes and clinical parameters by standardizing the median levels over time into z-scores, and examining each temporal slice to identify salient features. We related these features to survival outcomes using a multivariate Cox mixed-effect model to look for overall module effects, and separate univariate Cox models for each timepoint. Additional details can be found in Additional file 1.

## Results

### Clinical phenotyping

Main demographic characteristics of the study patient cohort are summarized in Table 1. Of 22 patients undergoing MCS-surgery, 5 patients died on the MCS device at 32 (20-50) days after surgery, where we report results in the usual format of median (interquartile range). The 17 survivors were followed until transplant (*n* = 13) for 107 (61–220) days or end of follow-up (December 31, 2014) (*n* = 4) for 600 (260–799) days. All non-survivors were male, and a higher fraction of non-survivors had an underlying ischemic etiology.

**Table 1:** Main characteristics of the study samples

	Characteristic	Total	Survivors	Non-survivors	*p*-value
Gender	Male	17	12	5	0.29
	Female	5	5	0	
Race	White or Caucasian	17	13	4	1
	Black or African American	2	2	0	
	Other	3	2	1	
Age	Median (IQR)	62.5 (42–66.5)	61 (38.5–65)	65 (62.5–76)	0.085
Etiology	Ischemic	6	2	4	0.009
	Non-Ischemic	16	15	1	
Diabetes	Diabetic	7	6	1	1
	Non-Diabetic	15	11	4	
INTERMACS	1 (Critical cardiogenic shock)	2	1	1	0.23
	2 (Progressive decline)	12	10	2	
	3 (Stable, but inotrope dependent)	7	6	1	
	4 (Resting symptoms)	1	0	1	
MCS Device	HeartMate II	13	11	2	0.25
	HeartWare	1	1	0	
	HeartMate II, CentriMag	4	2	2	
	TAH	1	1	0	
	PVAD	2	2	0	
	CentriMag BVAD	1	0	1	

**Fig. 2:**
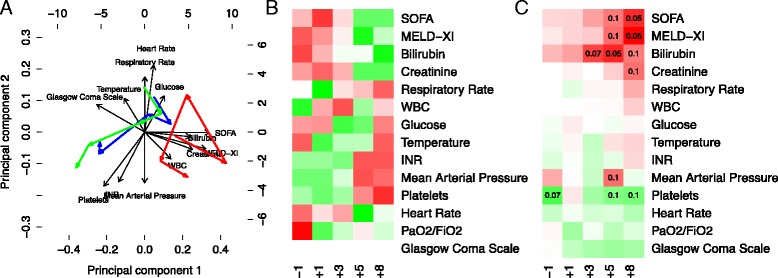
Analysis of clinical parameters. We analyzed the evolution of clinical parameters over time. (**a**) We projected the timepoint medians onto the leading principal components of the phenotype, and charted the median timecourse (blue) on a biplot. We then separated out the survivors (green) and non-survivors (red), and note a clear separation along the SOFA and MELD-XI dimensions. (**b**) We heatmapped median values at each timepoint. Each row is scaled to z-scores to bring out temporal contrasts, where red is upregulated and green is downregulated. On day 1 following surgery, we see the SOFA score peak, the platelet count, temperature, and respiratory rate trough. The white blood cell count reaches a maximum on day = +3, and then recovery occurs on day +5 and +8 as the platelet count rises and the SOFA score decreases. (**c**) We fit univariate Cox models for each clinical parameter at each timepoint, made a heatmap using signed –log *p* values (where the sign comes from the model coefficient), and displayed corrected q-values below q < 0.2. Notice that, with time, the organ dysfunction scores become highly predictive of survival (top right). Platelet count is also moderately predictive of survival, both at later points and before surgery

Principal component analysis revealed differences between survivors and non-survivors, and their clinical trajectories over time. In the biplot (Fig. 2a), clinically related parameters have a similar orientation vector, e.g. MELD-XI, SOFA, creatinine and bilirubin cluster together, forming an organ dysfunction axis along the first principal component. Additionally, anti-correlated variables have opposing orientations (e.g. GCS is antiparallel to the SOFA score, because the GCS is a descending scale where low scores indicate advanced organ dysfunction), and independent variables are orthogonal (e.g. heart rate, respiratory rate, and MAP appear to be somewhat independent of organ dysfunction, aligned with the second principal component). There is a clear separation between survivors and non-survivors along the SOFA and MELD-XI dimensions. This is expected, as non-survivors have overall worse organ dysfunction scores. However, the first timestep is towards increasing organ dysfunction in both survivors and non-survivors, indicating that all patients suffer from a decline in organ dysfunction in immediate response to surgery. Note that each subsequent timestep walks toward improving organ function in survivors yet not in non-survivors, who take a step back toward organ dysfunction by day +8.

We can construct a more detailed picture of the clinical trajectory by examining how parameters peak at different times (Fig. 2b). On the day following surgery, we see the SOFA score peak, while the platelet count, temperature, and respiratory rate trough. The white blood cell count reaches its maximum on day +3, and then recovery occurs on day +5 and +8 as the platelet count rises and the SOFA score decreases. All time-dependent features are summarized in Table 2.

**Table 2:** Summary of timecourse features

	Day −1	Day +1	Day +3	Day +5	Day +8
Upregulated phenotype	MELD-XI, bilirubin, temperature, PaO2/FiO2	SOFA, creatinine	WBC	MAP, INR, platelets	Platelets, temperature, INR, glucose, respiratory rate
Downregulated phenotype	WBC, MAP	Respiratory rate, temperature, platelets, MAP, PaO2/FiO2	Glucose, INR	MELD-XI, SOFA, creatinine, glucose, heart rate	SOFA, bilirubin, creatinine
Upregulated eigengene	Type I IFN, Transcription	Metabolic, mitochondria, innate	B cells, transcription	Mitosis, Defense, RNA processing	Apoptosis, protein folding, coagulation, catabolism
Downregulated eigengene	Mitosis, catabolism, defense, mitochondria, ribosome	T cells, demethylation, ER, transcription, coagulation, Type I IFN, NK cells	Type I IFN, NK cells	Innate	B cells, innate, metabolic, mitochondria

Finally, we discovered more detailed differences between survivors and non-survivors using a Cox model (Fig. 2c). We found that the organ dysfunction scores (SOFA and MELD-XI), driven by bilirubin and creatinine, became more predictive of survival at later timepoints. This is expected, as the survivors improve over time, but the non-survivors do not. We also see platelet count as a predictor of outcome both early and late in the timecourse. Survivors tended to increase platelet count during the recovery stage, while non-survivors did not. This matches clinical experience. Interestingly, platelet transfusion was required in certain patients, but did not seem to have a major effect closing the observed gap; we observed a relatively small change in platelet count for each unit replaced.

### Eigengene analysis

#### Eigengene network

**Fig. 3:**
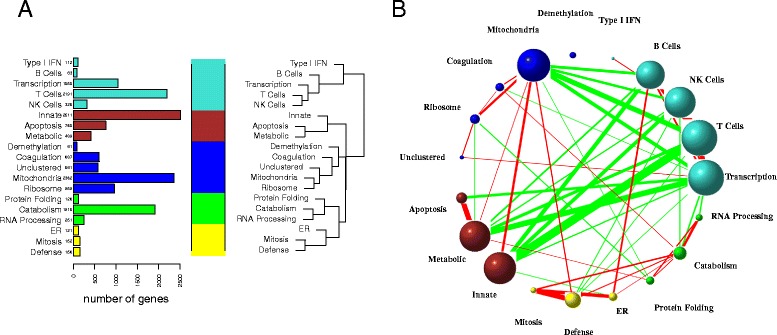
WGCNA network. (**a**) The WGCNA network has 19 modules, which we labeled using gene ontology enrichment analysis. To simplify the representation further, we hierarchically clustered the eigengenes to identify 5 superclusters, and color-coded them with distinct colors. Here, the turquoise supercluster can be understood as the adaptive immune system, the brown supercluster as components of the innate immune system, blue as a metabolic module, green as a catabolic module, and yellow as a reparative supercluster**.** The 4 most populated modules are T cells, catabolism, innate immunity, and mitochondria, all from separate superclusters. (**b**) WGCNA computes the eigengene network links using Pearson correlation, which we threshold at *r* > 0.3 for display. The color of each link indicates positive (red) or negative (green) correlation. The width of each link is proportional to the magnitude of correlation. The size of each node is proportional to the eigenvector centrality in the network. Notice the strong negative correlations between the innate and adaptive immune superclusters, the positive correlations of the mitochondria module with the innate module, and the negative correlations of the mitochondria module with the adaptive immune supercluster. The demethylation module is relatively disconnected, and is comprised mostly of Y-chromosome genes

We used WGCNA to infer a gene co-expression network consisting of 19 modules. We labeled the modules with biologically relevant terms using GO enrichment analysis (Fig. 3a), which we report in Additional file 1: Table S2. We also did pathway analyses for each module, and enrichment analyses of transcription factor binding sites, both of which are summarized in Additional file 1: Table S3. An in-depth discussion of the modules can be found in Additional file 1.

To assist in conceptualizing the eigengene network, we further looked for “superclusters” by clustering the eigengenes, and color-coded the result (Fig. 3a). Much of the structure we found can be explained succinctly: the turquoise supercluster can be understood as the adaptive immune system, the brown supercluster as components of the innate immune system, blue as metabolic, green as catabolic, and yellow as reparative. These supercluster colors are used throughout to aid in interpretation and organization.

Analysis of the cross-correlation between eigengenes revealed more detailed relationships in the network (Fig. 3b). The most distinctive feature is the strong negative correlation between the innate and adaptive immune superclusters, a feature that has been previously described in the literature [23]. We also found strong associations between these superclusters and the mitochondria module, suggesting a link between mitochondrial function and innate immunity. Mitochondria are recognized as principal mediators of inflammation and arbitrators of the pro-inflammatory state [30], have been characteristically studied in the context of organ dysfunction, and play a critical role in tissue hypoxia and production of reactive oxygen species [31–33]. Mitochondrial products have also been proposed as damage-associated molecular pattern (DAMP) signals [34] [35]. As we will see in the time-dependent analysis, the mitochondria and innate immune eigengenes peak together within the first 24 h after surgery. This coincidence suggests temporally orchestrated interactions between the two modules.

#### Mapping eigengenes to clinical parameters

**Fig. 4:**
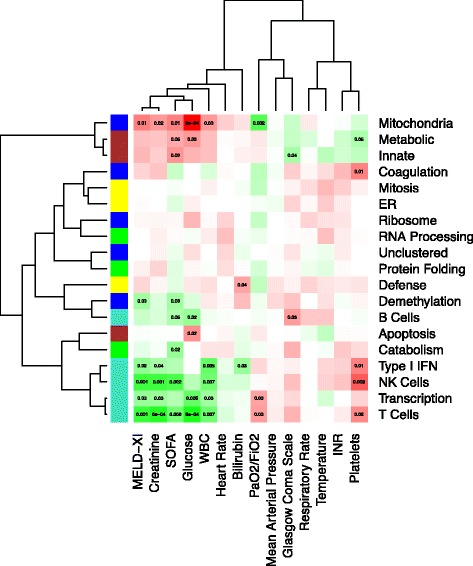
Mapping eigengenes to phenotypes. We inferred relationships between eigengenes and clinical parameters using a linear mixed-effect model, accounting for within-patient variance, and controlling for age, sex, race, diabetes, ischemic etiology, platelet transfusion, device type, plasmapheresis, and immunosuppression. We bi-clustered on the signed -log *p*-values to identify patterns, while displaying significant q-values. Note that the SOFA and MELD-XI scores are positively correlated with the innate (brown) and metabolic (blue) superclusters, and negatively correlated with the adaptive (turquoise) superclusters. Conversely, platelet count is positively correlated with the adaptive (turquoise) supercluster, while negatively correlated with the innate (brown) supercluster. Finally, the mitochondria module, which relates to both immunity superclusters, is associated with multiple indicators of organ failure

We uncovered relationships between the eigengenes and the clinical parameters using a linear mixed-effect model, and identified structure by bi-clustering the eigengenes with the clinical parameters (Fig. 4). Organ dysfunction, as measured by the SOFA and MELD-XI scores (along with creatinine, glucose, and white blood cell counts), is positively associated with the innate immunity, metabolic, and mitochondria modules. Conversely, organ dysfunction is negatively associated with the adaptive immunity supercluster, particularly the T cell, B cell, NK cell, Type I IFN, and transcription modules. This antagonistic pattern is consistent with previously reported innate activation and T-cell suppression [23], and believed to be causal: as innate-immunity-associated inflammation increases, organ dysfunction worsens, and the innate immune system suppresses the adaptive immune system. Conversely, as inflammation is suppressed and the adaptive immune system function returns, organ dysfunction improves.

We also found these three superclusters had significant associations with the platelet count, which is a parameter that contributes to the SOFA score and is predictive of survival. The platelet count had a positive association with the T cell, NK cell, type I IFN, and coagulation modules, and a negative association with the metabolic, innate, and mitochondria modules.

If we assume in concordance with published literature that 1) organ dysfunction is mediated by specific interaction patterns between leukocytes, EC and PLT, 2) more innate inflammation is associated with “more sticky” EC and PLT, and 3) more PLT-clot leads to more PLT-consumption, our transcriptome-phenome data point towards an orchestrated system-wide behavior that either “cools down” to allow recovery and survival or further escalates “out of control” towards death.

Finally, we found an association between glucose levels and organ dysfunction scores, which is also consistent with the literature. Higher glucose levels have been associated, likely via increased adreno-cortical drive, with an increased 30-day mortality rate in conditions such as acute heart failure [36, 37].

#### Time-dependent eigengene analysis

We expected to see distinctive time-dependent features in the transcriptome, because the surgery is a significant perturbation to the system that is already characterized by AdHF-induced changes, and the recovery is very dynamic. As with the clinical parameters, we characterized these features by examining how the median eigengene levels changed over time.

**Fig. 5:**
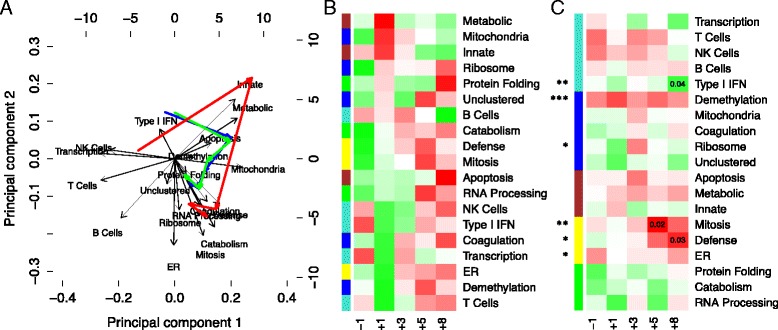
Time-dependent eigengene analysis. We analyzed the evolution of eigengene levels over time. (**a**) We projected the timepoint medians onto the leading principal components of the eigengene expression, and charted the median timecourse (blue) on a biplot. We then separated the survivors (green) and non-survivors (red), revealing an exaggerated innate immune and metabolic first step in non-survivors. (**b**) We made a heatmap of the median values for each eigengene at each timepoint. Each row is scaled to z-scores to bring out temporal contrasts, where red is upregulated and green is downregulated. Here, we ordered the rows by sorting z-scores at day 1. We found a response to surgery orchestrated into stages: first, activation of the innate immune response, followed by anti-inflammation, and finally reparative processes such as mitosis, coagulation, and apoptosis. (**c**) To infer which eigengenes are related to survival outcomes, we fit a multivariate mixed-effects Cox model to the eigengenes (asterisks), and univariate Cox models at each timepoint (uncorrected *p*-values displayed), and made a heatmap using signed –log *p* values (where the sign comes from the model coefficient). Note that the reparative (yellow) supercluster emerges as important in the last two timepoints, along with the Type I IFN module. The mitosis and defense modules, marked by significant *p*-values, are related to cellular regeneration and extracellular matrix (ECM) degradation, and suggest organ failure due to disseminated coagulopathy

Principal component analysis revealed differences between survivors and non-survivors, and their eigengene trajectories over time. In the biplot (Fig. 5a), the orientation of the eigengene vectors reflects the superclustering we have previously noted, where the innate and adaptive immune system are antiparallel, with the remaining eigengenes acting orthogonally to both. We notice a net displacement from day −1 to day 8 in the direction of the reparative and catabolic superclusters, indicative of the recovery processes activated following the surgery for all patients. The second timepoint, however, is a large deviation from that trajectory, which we associate with activation of the innate immunity supercluster, and suppression of the adaptive immunity supercluster. This deviation is much more pronounced in the non-survivors.

We uncovered a more detailed picture of the eigengene trajectory by noting how eigengenes peak at different times (Fig. 5b). We found a genomic response to surgery orchestrated into stages: first, activation of the innate immune response, followed by anti-inflammation, and finally reparative processes such as mitosis, coagulation, and apoptosis. These temporal features are summarized in Table 2, and a more in-depth discussion can be found in Additional file 1.

Finally, we discovered more detailed differences between survivors and non-survivors using a Cox model (Fig. 5c). We found a consistent effect from the demethylation module across all timepoints, but suspect this result is artifactual. The demethylation module is heavily enriched by Y-chromosome genes, indicating that the effect is likely due to the fact that all non-survivors were male. We also found mitosis and defense eigengenes becoming predictive late in the timecourse. These features are related to cellular regeneration and extracellular matrix (ECM) degradation, and are consistent with organ failure due to disseminated intravascular coagulopathy (DIC). They are also consistent with the failure of non-survivors to recover platelet count late in the time course. A more detailed discussion of these modules can be found in Additional file 1.

## Discussion

Providing the best possible care for AdHF patients requiring advanced therapies such as MCS-surgery as bridge to recovery, bridge to transplantation or destination therapy is an important challenge for modern medicine. The 1-year mortality rate following MCS implantation is relatively high compared to other medical procedures, in the range of 20% [1]. A better understanding and prediction of clinical outcomes requires a model that accurately captures a multitude of complex features in the systemic inflammatory response. In this context, the main results of this study 1) demonstrate a clear phenome (organ dysfunction)/PBMC-transcriptome-relationship, 2) support a time-dependent evolution of the PBMC-transcriptome as it relates to survival/non-survival outcomes, and 3) allow to generate a hypothesis of the role of coagulation-and platelet-related biology in the survival fate of the AdHF-patient after MCS-surgery.

The necessity of developing an appropriate conceptual immunological framework has been stated by various groups [31, 38], but has been accompanied by significant challenges. Genome-wide transcription profiling in human sepsis has indicated that both pro- and anti-inflammatory mechanisms occur at various times over the course of sepsis [39, 40], that a patient may cycle through each phase multiple times [41] and that the transition from infection with pathogen clearance over sepsis with organ failure to septic shock with death [42] calls for a unifying integrated hypothesis linking inflammation, energy metabolism, coagulopathy and organ dysfunction by studying the interaction between leukocytes, endothelial cells and platelets in a phenotype- and time-dependent design. There are fundamental problems for the classical biphasic model [31], which assumes a systemic inflammatory response syndrome (SIRS) followed by a compensatory anti-inflammatory response syndrome (CARS) [43]. Still, recent investigations continue to build upon this limited model, stating that the biphasic view “may be a simplistic explanation of a complex disease, yet provides a rational explanation for how the function of the immune system becomes altered during the course of sepsis” [44]. An integrative model that moves beyond the current SIRS/CARS framework would be of great use to the study of MCS- related MOD.

In this study, we developed an integrative model by utilizing a systems biological approach, based on three research study design assumptions. First, we take the clinical phenotypic trajectory as the most reliable, relevant, and authoritative framework for modeling molecular data in a hypothesis-agnostic and discovery-driven way. Second, we assume that a systems approach, linking the longitudinal time-series clinical phenotype to the entire transcriptome, is the least reductionist approach to developing a unified model of inflammation in MOD [31, 44]. And third, we assert that an integrative model provides the best framework for generating and interpreting novel integrative mechanistic hypotheses regarding pathophysiology, diagnosis, prognosis and treatment of heart failure.

Integrated research strategies have previously been suggested by other groups [35, 45] and successfully implemented by our own [20, 21]. By adopting this strategy, we integrated the current biphasic SIRS/CARS-model into a more unbiased and comprehensive framework. The distinctive, previously published immunological features of SIRS/CARS [46] are well-captured by our model at day 1 and 3, when we note the prominent innate immune response and its subsequent suppression and transition to adaptive immunity (Fig. 5b). However, our model has the advantage of incorporating several features that the SIRS/CARS model does not readily accommodate. We note a number of eigengenes that act orthogonally to the innate/adaptive immune response (Fig. 5a), which we functionally classify as metabolic, catabolic, and reparative processes [32]. Recovery mechanisms emerge as distinctive features in our model, and are particularly interesting at later timepoints when the SIRS/CARS framework is no longer helpful (Fig. 5b and c). In this way, we functionally characterized distinct stages of recovery (or non-recovery) from MCS surgery, unifying a number of disparate hypotheses. The result is clinically relevant to the goal of improving prediction of adverse events and designing early pre-emptive therapeutic interventions.

**Fig. 6:**
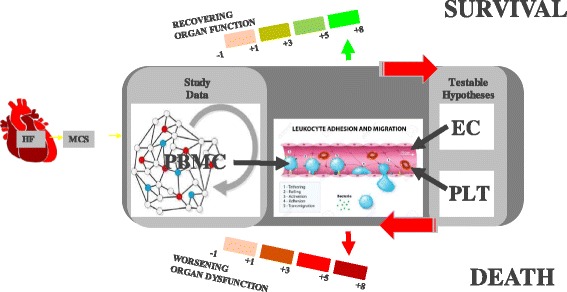
Hypothesis Linking PBMC-Mediated Inflammation, Organ Dysfunction and Death: In heart failure (**HF**)-patients requiring mechanical circulatory support (**MCS**) surgery, the time-dependently activated and well orchestrated innate inflammatory activation (as measured by time-series **PBMC**-transcriptome data and hypothetical interaction with endothelial cells (**EC**) and platelets (**PLT**)) and related organ dysfunction either serves to restore improved oxygen delivery, “cooling down” of inflammation, normalization of organ function, recovery and survival, or escalates and leads to death

The data-driven interpretation of our study results, based on the above assumptions of the clinical phenotypic trajectory as the most reliable, relevant, and authoritative framework and longitudinal time-series approach needs to drill deep into the source data: In relation to the ultimate endpoint of being alive or dead at the end of followup, each timepoints phenotype data have predictive information (more advanced organ dysfunction predicing a higher likelihood of death). Hypothesizing that our PBMC transcriptome data improve this prediction, our transcriptome data interpretation must necessarily be phenotype- and time-dependent*.* The phenotype- and time-dependent module eigengenome interpretation must accept the systems-theoretical “downward-causation” [47] property of the time-dependent phenotype onto the PBMC-module eigengenome. This interpretative strategy leads us to the following insights:

1. Attempts of a phenotype- and time-independent interpretation of the PBMC-transcriptome have to be resisted in order to be effective. This insight may explain why many of the current critical care interventions aimed at reducing risk of dying in MOD have so far proven unsuccessful.
2. An iterative algorithmized approach to predict future phenotype (survival versus death), based on the time-dependent phenotype (summed up over all preceding timepoints, refined by the time—and phenotype-dependent module-eigenegenome) seems feasible and is a logical strategic step.
3. Higher SOFA and MELD-XI score on timepoints 4 and 5 and higher mitosis and defense eigengene expression on timepoints 4 and 5 are associated with worse subsequent survival (as indicator of biological need to counteract destabilizing trends), while higher platelet scores on timepoints 1, 4, 5 and higher IFN-eigengene expression (as indicator of adaptive immunity recovery) on timepoint 5 are associated with better subsequent survival.
4. The following integrated hypothesis emerges: AdHF- and MCS-induced innate non-specific inflammatory response is causally linked in various/all organs – via EC- and PLT-interactions – with energy-inefficiency (mitochondrial module) and coagulopathy with low PLT-count and organ dysfunction, likely via microvascular clot formation. If the pattern reverses (indicated by improvement of the SOFA-score, MELD-XI score, PLT, adaptive immunity modules, the patient’s probability of survival increases. If it does not reverse, the patient’s probability of death increases.
5. We can now hypothesize that in a milieu of chronic activation of the SNS and RAAS (first level of compensation) as well as varying degrees of activation of the immune system (second level of compensation), the implantation of the longterm MCS (third level of compensation) (Fig. 1) adds an unpredictable perturbation to the system. This added perturbation with its time-dependently activated and well orchestrated innate inflammatory and procoagulatory activation (as measured by time-series PBMC-transcriptome data) and related organ dysfunction either serves to restore improved oxygen delivery, “cooling down” of inflammation, resolution of coagulopathy, normalization of organ function, recovery and survival, or escalates and leads to death (Fig. 6).

## Limitations

### Control population

While we chose to base this analysis on AdHF-patients undergoing MCS-surgery alone to address to most pressing clinical problem of MCS-related perioperative MOD [1, 7, 8], we acknowledge that we have not addressed the question which aspects of the PBMC-biology addressed here is specific to the MCS-surgery intervention versus related to heart surgery in general. In order to address this important question, we have initiated a followup project examining AdHF-cohorts undergoing A) Optimal Medical Management, B) Heart transplantation, C) High Risk Coronary Artery Bypass Surgery and D) healthy volunteers, utilizing the same study protocol.

### Biological validation studies

This study was designed as a hypothesis-generating study. In our ongoing work, we are generating biological validation data.

### Sample size

While our model was able to accurately identify and integrate many known features of organ dysfunction following MCS surgery, we acknowledge statistical limitations due to the small sample size of our dataset. The small sample size affects the predictive modeling aspect of our study, most particularly the Cox survival models, while the unsupervised approach based on WGCNA has been shown in previous studies to be robust even at small sample sizes (*n* < 30) [48–50]. Furthermore, because of our repeated-measures design, the number of total samples used was sufficiently large when inferring the eigengene network, and when relating it to the clinical parameters using a linear mixed-effect model. Thus, most of the statistical limitations in this experiment arise from the heterogeneity of the small patient cohort, rather than the inferential methods used in analysis, which is a limitation that can only be addressed by expanding our scope to a coordinated multi-center study to gain a much larger sample size which is currently being implemented.

## Conclusions

Our model identifies and synchronizes, at an interpretable level of detail, the many interesting clinical and biological features in the systemic inflammatory response following MCS device implantation. It provides an integrative systems biological model, spanning the full PBMC leukocyte genome, while depicting an orchestrated molecular trajectory. This biological sequence of events acts to functionally characterize distinct stages of recovery from MCS surgery, and can help in understanding progressive worsening. This model is also clinically relevant to exploring and understanding pre-emptive therapeutic interventions, and to improving prediction of adverse events.

## Additional file

Additional file 1: Supplemental Analysis. (DOCX 12807 kb)

## Abbreviations

AdHF: Advanced heart failure
BVAD: Biventricular assist device
CARS: Compensatory anti-inflammatory response syndrome
CO: Cardiac output
CPT: Cell Preparation Tubes
DAMP: Damage-associated molecular pattern
DIC: Disseminated intravascular coagulopathy
EC: Endothelial cell
ECM: Extracellular matrix
FiO2: Fraction of inspired oxygen
GCS: Glasgow Coma Scale
GO: Gene ontology
INR: International Normalized Ratio
INTERMACS: Interagency Registry for Mechanically Assisted Circulatory Support
MAP: Mean systemic arterial pressure
MCS: Mechanical circulatory support
MELD-XI: Model of Endstage Liver Disease without except International Normalized Ratio (INR)
MOD: Multiple Organ Dysfunction Syndrome
O2: Oxygen delivery
PBMC: Peripheral blood mononuclear cell
PLT: Platelet
PVAD: Paracorporeal Ventricular Assist Device
RAAS: Renin-angiotensin-aldosterone system
SIRS: Systemic inflammatory response syndrome
SNS: Sympathetic nervous system
SOFA: Sequential Organ Failure Assessment score
t-TAH: Temporary Total Artificial Heart
WGNCA: Weighted gene co-expression network analysis

## References

[1] Kirklin JK, Naftel DC, Pagani FD, Kormos RL, Stevenson LW, Blume ED, Miller MA, Timothy Baldwin J, Young JB (2014). Sixth INTERMACS annual report: a 10,000-patient database. The Journal of heart and lung transplantation : the official publication of the International Society for Heart Transplantation.

[2] Deng MC, Naka Y (2007). Mechanical circulatory support therapy in ADVANCED HEART FAILURE.

[3] Zhang Q, Raoof M, Chen Y, Sumi Y, Sursal T, Junger W, Brohi K, Itagaki K, Hauser CJ (2010). Circulating mitochondrial DAMPs cause inflammatory responses to injury. Nature.

[4] Meerson FZ (1962). Compensatory hyperfunction of the heart and cardiac insufficiency. Circ Res.

[5] Bone RC, Balk RA, Cerra FB, Dellinger RP, Fein AM, Knaus WA, Schein RM, Sibbald WJ (1992). Definitions for sepsis and organ failure and guidelines for the use of innovative therapies in sepsis. The ACCP/SCCM consensus conference committee. American College of Chest Physicians/Society of Critical Care Medicine. Chest.

[6] Levine B, Kalman J, Mayer L, Fillit HM, Packer M (1990). Elevated circulating levels of tumor necrosis factor in severe chronic heart failure. N Engl J Med.

[7] Deng MC, Loebe M, El-Banayosy A, Gronda E, Jansen PG, Vigano M, Wieselthaler GM, Reichart B, Vitali E, Pavie A, Mesana T, Loisance DY, Wheeldon DR, Portner PM (2001). Mechanical circulatory support for advanced heart failure: effect of patient selection on outcome. Circulation.

[8] Deng MC, Edwards LB, Hertz MI, Rowe AW, Keck BM, Kormos R, Naftel DC, Kirklin JK, Taylor DO, International Society for H, Lung T (2005). Mechanical circulatory support device database of the International Society for Heart and Lung Transplantation: third annual report--2005. J Heart Lung Transplant.

[9] Bone RC (1996). Immunologic dissonance: a continuing evolution in our understanding of the systemic inflammatory response syndrome (SIRS) and the multiple organ dysfunction syndrome (MODS). Ann Intern Med.

[10] Fry DE (2012). Sepsis, systemic inflammatory response, and multiple organ dysfunction: the mystery continues. Am Surg.

[11] Rittirsch D, Flierl MA, Ward PA (2008). Harmful molecular mechanisms in sepsis. Nat Rev Immunol.

[12] Brown KA, Brain SD, Pearson JD, Edgeworth JD, Lewis SM, Treacher DF. Neutrophils in development of multiple organ failure in sepsis. *Lancet (London, England)* 2006;368:157–169. 10.1016/S0140-6736(06)69005-3 16829300

[13] Levi M, van der Poll T, Schultz M (2012). Systemic versus localized coagulation activation contributing to organ failure in critically ill patients. Semin Immunopathol.

[14] Aird WC (2003). The role of the endothelium in severe sepsis and multiple organ dysfunction syndrome. Blood.

[15] Feldman D, Pamboukian SV, Teuteberg JJ, Birks E, Lietz K, Moore SA, Morgan JA, Arabia F, Bauman ME, Buchholz HW, Deng M, Dickstein ML, El-Banayosy A, Elliot T, Goldstein DJ, Grady KL, Jones K, Hryniewicz K, John R, Kaan A, Kusne S, Loebe M, Massicotte MP, Moazami N, Mohacsi P, Mooney M, Nelson T, Pagani F, Perry W, Potapov EV, Eduardo Rame J, Russell SD, Sorensen EN, Sun B, Strueber M, Mangi AA, Petty MG, Rogers J (2013). The 2013 International Society for Heart and Lung Transplantation guidelines for mechanical circulatory support: executive summary. J Heart Lung Transplant.

[16] Adigopula S, Vivo RP, DePasquale EC, Nsair A, Deng MC (2014). Management of ACCF/AHA stage C heart failure. Cardiol Clin.

[17] DENG MC (2007). NAKA Y.

[18] Vincent JL, Moreno R, Takala J, Willatts S, De Mendonca A, Bruining H, Reinhart CK, Suter PM, Thijs LG (1996). The SOFA (sepsis-related organ failure assessment) score to describe organ dysfunction/failure. On behalf of the working group on sepsis-related problems of the European Society of Intensive Care Medicine. Intensive Care Med.

[19] Kamath PS, Wiesner RH, Malinchoc M, Kremers W, Therneau TM, Kosberg CL, D'Amico G, Dickson ER, Kim WR. A model to predict survival in patients with end-stage liver disease. *Hepatology (Baltimore, Md)* 2001;**33**:464–470. 10.1053/jhep.2001.22172 11172350

[20] Deng MC, Eisen HJ, Mehra MR, Billingham M, Marboe CC, Berry G, Kobashigawa J, Johnson FL, Starling RC, Murali S, Pauly DF, Baron H, Wohlgemuth JG, Woodward RN, Klingler TM, Walther D, Lal PG, Rosenberg S, Hunt S (2006). Noninvasive discrimination of rejection in cardiac allograft recipients using gene expression profiling. Am J Transplant Off J Am Soc Transplant Am Soc Transplant Surg.

[21] Pham MX, Teuteberg JJ, Kfoury AG, Starling RC, Deng MC, Cappola TP, Kao A, Anderson AS, Cotts WG, Ewald GA, Baran DA, Bogaev RC, Elashoff B, Baron H, Yee J, Valantine HA (2010). Gene-expression profiling for rejection surveillance after cardiac transplantation. N Engl J Med.

[22] Bondar G, Cadeiras M, Wisniewski N, Maque J, Chittoor J, Chang E, Bakir M, Starling C, Shahzad K, Ping P, Reed E, Deng M (2014). Comparison of whole blood and peripheral blood mononuclear cell gene expression for evaluation of the perioperative inflammatory response in patients with advanced heart failure. PLoS One.

[23] Sinha A, Shahzad K, Latif F, Cadeiras M, Von Bayern MP, Oz S, Naka Y, Deng MC (2010). Peripheral blood mononuclear cell transcriptome profiles suggest T-cell immunosuppression after uncomplicated mechanical circulatory support device surgery. Hum Immunol.

[24] Leek JT, Johnson WE, Parker HS, Fertig EJ, Jaffe AE, Storey JD. Surrogate Variable Analysis. Bioconductor version: Release (31) 2015.

[25] Tritchler D. Corresponding, Parkhomenko E, Beyene J. Filtering genes for cluster and network analysis. BMC Bioinformatics. 2009;10 10.1186/1471-2105-10-193 PMC2708160 19549335

[26] Langfelder P, Horvath S (2008). WGCNA: an R package for weighted correlation network analysis. BMC Bioinformatics.

[27] ¨hlich HF. The GOSim package. 2015.

[28] Pvt.Ltd. SLS. Strand NGS Manual. 2.1 ed. San Francisco: Strand Genomics, Inc., 2014.

[29] Loots G, Ovcharenko I. rVista 2.0: evolutionary analysis of transcription factor binding sites. Nucleic Acids Research: 32(Web Server Issue), 2004. 10.1093/nar/gkh383 PMC441521 15215384

[30] Strowig T, Henao-Mejia J, Elinav E, Flavell R (2012). Inflammasomes in health and disease. Nature.

[31] Deutschman CS, Tracey KJ (2014). Sepsis: current dogma and new perspectives. Immunity.

[32] Singer M (2014). The role of mitochondrial dysfunction in sepsis-induced multi-organ failure. Virulence.

[33] Dare AJ, Phillips AR, Hickey AJ, Mittal A, Loveday B, Thompson N, Windsor JA (2009). A systematic review of experimental treatments for mitochondrial dysfunction in sepsis and multiple organ dysfunction syndrome. Free Radic Biol Med.

[34] van Kempen TS, Wenink MH, Leijten EF, Radstake TR, Boes M. Perception of self: distinguishing autoimmunity from autoinflammation. Nat Rev Rheumatol. 2015; 10.1038/nrrheum.2015.60 25963881

[35] Langley RJ, Tsalik EL, van Velkinburgh JC, Glickman SW, Rice BJ, Wang C, Chen B, Carin L, Suarez A, Mohney RP, Freeman DH, Wang M, You J, Wulff J, Thompson JW, Moseley MA, Reisinger S, Edmonds BT, Grinnell B, Nelson DR, Dinwiddie DL, Miller NA, Saunders CJ, Soden SS, Rogers AJ, Gazourian L, Fredenburgh LE, Massaro AF, Baron RM, Choi AM, Corey GR, Ginsburg GS, Cairns CB, Otero RM, Fowler VG, Jr., Rivers EP, Woods CW, Kingsmore SF. An integrated clinico-metabolomic model improves prediction of death in sepsis. Sci Transl Med 2013;**5**:195ra195. 10.1126/scitranslmed.3005893 PMC3924586 23884467

[36] Mebazaa A, Gayat E, Lassus J, Meas T, Mueller C, Maggioni A, Peacock F, Spinar J, Harjola VP, van Kimmenade R, Pathak A, Mueller T, Tavazzi L, Disomma S, Metra M, Pascual-Figal D, Laribi S, Logeart D, Nouira S, Sato N, Parenica J, Deye N, Boukef R, Collet C, Van den Berghe G, Cohen-Solal A, Januzzi JL (2013). Association between elevated blood glucose and outcome in acute heart failure: results from an international observational cohort. J Am Coll Cardiol.

[37] Losser MR, Damoisel C, Payen D (2010). Bench-to-bedside review: Glucose and stress conditions in the intensive care unit. Crit Care.

[38] Vaz NM, Carvalho CR (2015). On the origin of immunopathology. J Theor Biol.

[39] Cavaillon JM, Annane D (2006). Compartmentalization of the inflammatory response in sepsis and SIRS. J Endotoxin Res.

[40] Tang BM, Huang SJ, McLean AS (2010). Genome-wide transcription profiling of human sepsis: a systematic review. Crit Care.

[41] Hotchkiss RS, Monneret G, Payen D (2013). Immunosuppression in sepsis: a novel understanding of the disorder and a new therapeutic approach. Lancet Infect Dis.

[42] Bhan C, Dipankar P, Chakraborty P, Sarangi PP (2016). Role of cellular events in the pathophysiology of sepsis. Inflammation research : official journal of the European Histamine Research Society [et al].

[43] Faix JD (2013). Biomarkers of sepsis. Crit Rev Clin Lab Sci.

[44] Boomer JS, Green JM, Hotchkiss RS (2014). The changing immune system in sepsis: is individualized immuno-modulatory therapy the answer?. Virulence.

[45] Ravasz E, Somera AL, Mongru DA, Oltvai ZN, Barabasi AL. Hierarchical organization of modularity in metabolic networks. *Science (New York, NY)* 2002;297:1551–1555. 10.1126/science.1073374 12202830

[46] Cobb JP, O'Keefe GE. Injury research in the genomic era. *Lancet (London, England)* 2004;363:2076–2083. 10.1016/S0140-6736(04)16460-X 15207961

[47] Raia F (2012). Mechanisms, causality, and explanations in complex geodynamic systems. The Geological Society of America.

[48] Gong KW, Zhao W, Li N, Barajas B, Kleinman M, Sioutas C, Horvath S, Lusis AJ, Nel A, Araujo JA (2007). Air-pollutant chemicals and oxidized lipids exhibit genome-wide synergistic effects on endothelial cells. Genome Biol.

[49] Miller JA, Oldham MC, Geschwind DH (2008). A systems level analysis of transcriptional changes in Alzheimer's disease and normal aging. The Journal of neuroscience : the official journal of the Society for Neuroscience.

[50] Gargalovic PS, Imura M, Zhang B, Gharavi NM, Clark MJ, Pagnon J, Yang WP, He A, Truong A, Patel S, Nelson SF, Horvath S, Berliner JA, Kirchgessner TG, Lusis AJ (2006). Identification of inflammatory gene modules based on variations of human endothelial cell responses to oxidized lipids. Proc Natl Acad Sci U S A.

