# Supplementary material for: Integrative model of leukocyte genomics and organ dysfunction in heart failure patients requiring mechanical circulatory support: a prospective observational study
Source: BMC Med Genomics. 2017 Aug 29;10:52. doi: 10.1186/s12920-017-0288-8 (PMC5576384; doi:10.1186/s12920-017-0288-8)
Supplement: Additional file 1: — Supplemental Analysis. (DOCX 12807 kb) [file 12920_2017_288_MOESM1_ESM.docx]

# Additional file 1

## Organ dysfunction scoring systems

### Sequential Organ Failure Assessment (SOFA) Score

The SOFA score is a simple, validated, and widely accepted measure that can be easily obtained using the above parameters, and is frequently used to assess disease severity. It has been shown to be predictive of survival in the critical care unit [^18^](#_ENREF_18), and has been applied to different study populations. The SOFA score is an integer scoring system that assigns a numerical variable to each of 6 major organ systems to quantify the severity of organ failure. Values range between 0 and 4. Each system’s value is summed into a single SOFA score. Therefore the sum score ranges between 0 and 24, and positively correlates with the severity of the MOD syndrome and clinical outcomes.

Per clinical protocol at our academic center, only those parameters used for clinical assessment were sampled, and only those drugs used for clinical management were included in the database for the study. Therefore, we used the approximate estimation to the SOFA score using the following criteria (Table S1). The presence or absence of inotropic and vasoactive drugs varies with the SOFA score.

The cardiovascular section of the SOFA score was modified due to certain limitations. Inotropic drugs were presented in the electronic medical records in several different dosage units. Therefore, there was no clear method of comparing the drug doses onto a same scale, and we made the following adjustments:

0: Mean arterial pressure greater than 70, No Drugs

1: Mean arterial pressure less than 70, No Drugs

2: Mean arterial pressure less than 70, 1 drug, at low dose

3: Mean arterial pressure less than 70, 1 or more drugs on intermediate doses

4: Mean arterial pressure less than 70, 1 or more drugs on high doses

The respiratory parameter (PaO2/FiO2 ratio) was frequently missing due to intubation. We therefore made the following replacements in computing SOFA: patients not intubated were given a 1, and patients on respiration support were given a 3.

The neurological parameter (Glasgow Coma Scale (GCS)) was frequently missing from electronic medical charts, due to patients typically being sedated. GCS is a scoring system between 3 and 15 (with 3 being worst, and 15 being best) and is used to determine the conscious status of a patient. It is composed of three parameters: eye response, verbal response, and motor response [^51^](#_ENREF_51). Patients are supposed to receive the following SOFA score contributions based on the Glasgow Coma Rating:

0: GCS = 15

1: GCS = 13-14

2: GCS = 10-12

3: GCS = 6-9

4: GCS <6

However, patients who are sedated were assigned GCS scores of 3 (worst), based on the Ramsay Sedation Scale.

### Model for End-stage Liver Disease except INR (MELD-XI) score

The MELD-XI score incorporates both bilirubin and creatinine, and is defined [^52^](#_ENREF_52)^,^ [^53^](#_ENREF_53) by MELD-XI = 5.11 Ln(bilirubin) + 11.76 Ln(creatinine) + 9.44.

## Statistical methods

### Principal Component Analysis

To characterize a typical eigengene time course following the surgery, we looked at median eigengene values. Visualization was done using a heatmap (‘heatmap.2’ R package) and PCA biplot (‘biplot’, ‘pca’ R packages). Salient temporal features were found by scaling the medians about their temporal aspect, converting the median eigengene levels to standard z-scores. At each timepoint, module relevance was inferred by then sorting the median eigengenes according to z-score, with the most salient features having the most extreme z-scores.

### WGCNA

To construct a systems representation, we inferred a weighted co-expression network using the WGCNA package in R [^26^](#_ENREF_26). WGCNA efficiently approximates a network adjacency matrix by starting from the cross-correlation matrix. While our dataset has a repeated measures structure, we ignored mixed effects modeling at this step for the sake of computational efficiency, incorporating all samples into the calculation regardless of time or group label. We used the absolute Pearson correlation as an adjacency measure, because we wanted to cluster without regard to the sign of the correlation, in order to improve interpretability under enrichment analyses. WGCNA next alters the adjacency matrix to become approximately scale-free, by raising each element to the smallest exponent that sufficiently maximizes a scale-free fit. The motivation here lies with the assumption that biological networks are approximately scale-free networks [^45^](#_ENREF_45). Finally, topological overlap information is used to improve the reliability of the adjacency matrix.

The adjacency matrix was next partitioned by hierarchical clustering, and a dynamic tree-cutting algorithm was used to optimize module assignment. With each module consisting on average of several hundred genes, dimensionality was further reduced using principal component analysis to compute a representative eigengene summarizing the expression of an entire module in a single vector. We computed eigengenes for each module, and used the correlation between eigengenes to define an eigengene network.

### Linear Mixed-Effect Model

To relate the modules to the clinical phenotypes, we used a linear mixed-effect model [^54^](#_ENREF_54). By using a mixed-effect model, we account for the repeated-measures structure of the data. The statistical significance of the mixed model was corrected for multiple testing using the ‘fdrtools’ R package [^55^](#_ENREF_55).

### Bioinformatics

We computed gene ontology enrichments for each module using the ‘GOsim’ R package [^56^](#_ENREF_56) (Table S2). We conducted a pathway analysis on each module using Strand NGS bioinformatics software [^28^](#_ENREF_28). Module gene lists were analyzed with a library of “Legacy” pathways from Strand NGS’s Database. We selected the top 5 pathways based on number of entities matched and the p-value. We then used Strand’s natural language processing (NLP) algorithm to identify “pathway hubs,” or the most highly connected genes within each pathway. This algorithm uses IntAct and PubMed abstracts to extract gene interactions, and rank genes based on global connectivity and a relation score. Additionally, we analyzed each module for enrichment of transcription factor binding sites using the whole genome rVista tool [^29^](#_ENREF_29). The top genes, pathways, and transcription factor binding sites of interest are indicated in Table S3**.**

### Cox Proportional Hazards Model

To relate the modules to survival outcomes, we used a Cox mixed-effect model, the ‘coxme’ R package [^57^](#_ENREF_57). However, we anticipated significant dynamic effects that could lead to problems with a single model for all timepoints, and also fit separate Cox models at each timepoint using the ‘survival’ R package [^58^](#_ENREF_58). When creating the final model, we used the elastic-net implementation in the ‘glmnet’ R package [^59^](#_ENREF_59).

### Cox p-value Distributions per Module

When working with unsupervised methods such as WGCNA, there is always concern that the eigengenes may be too great an abstraction, and that important statistical properties have been compressed away. To verify that the eigengene model is consistent with the statistical properties of the genes themselves, we computed models for each individual gene, analogous to our analysis of eigengenes: we used a linear mixed-effect model to infer relationships between genes and clinical parameters, and we used a Cox model at each timepoint to infer associations between genes and survival outcomes. We then tested the hypothesis that predictive eigengenes are associated with predictive genes. A hypothesis of no association implies that the distribution of p-values within a module should be flat, while a true association will exhibit an abundance of statistically significant genes that skew the p-distribution. To quantify this hypothesis test, we analyzed the distribution of p-values for genes in each module using a binomial test, performed by dichotomizing around the usual significance threshold *p*=0.05.

## Gene Module Discussion

Here we present a detailed discussion of our investigation into every module’s role in the systems biology of the systemic inflammatory response, organized by superclusters and their putative role in mediating clinical organ dysfunction in AdHF patients undergoing MCS therapy. Our data are interpreted in the clinical-translational framework of AdHF-pathophysiology (**Figures 1,4 and 6**):

### Innate immune supercluster

#### Innate

The innate immune system initially responds to the surgical intervention by the activation of a variety of immune cells including basophils, neutrophils, macrophages and eosinophils. It generally provides a rapid response to a pathogen by activation of toll-like receptors (TLRs) via pathogen-associated molecular pattern molecules (PAMPs), or damage-associated molecular pattern molecules (DAMPs), which are cell-derived and initiate and perpetuate immunity in response to trauma, ischemia, and tissue damage [^40^](#_ENREF_40). In our pathway analysis of this module, hubs involved in the enriched pathways include inflammatory cytokines such as IL-1 and TNF-α. The activation of these cytokines results in activation of signal transduction pathways (like RAC1), stimulating further production of cytokines, procoagulants, ROS, and proliferative factors.

As expected, the innate module spikes on day 1 after surgery, initiating the inflammatory response (**Figure 5B**). This is associated with worsening organ dysfunction, as measured by the SOFA and MELD-XI scores (**Figure 4**). Worse acute and chronic hepatic and renal function has been described in association with proinflammatory cytokines [^60^](#_ENREF_60). The innate module is also associated with higher blood glucose levels, which is not surprising given the overlap with the mitochondria module. It is also associated with higher white blood cell count (**Figure 4**), which has a delayed peak on day 3 (**Figure 2B**). Although the Cox model was not statistically significant at any timepoint, the trend of the coefficients suggests a more extreme activation on day 1, and a more extreme supression on day 3, in non-survivors compared to survivors (**Figure 5C**).

#### Apoptosis

The apoptosis module is correlated with the metabolic module, and anti-correlated with the transcription module (**Figure 3**). Like the innate, metabolic, and mitochondria modules, it is associated with higher blood glucose (**Figure 4**). It is enriched for signaling and cytokine activity, indicating that mitochondrial apoptotic pathways are being activated by pro-inflammatory cytokines. These pathways are known to play in important role in organ dysfunction, because the termination of the inflammatory response is effected through apoptosis ^[61](#_ENREF_61)^. In patients with sepsis, neutrophil apoptosis is inversely proportional to the severity of organ dysfunction ^[62](#_ENREF_62)^. We further observe that the module is associated with granulocyte specific cell markers, and shows a strong (*p*=8.4E-9) enrichment for genes associated with decreased susceptibility to endotoxin shock, suggesting that this module may be regulating the above pathway. We found it inhibited through most of the timecourse, and upregulated primarily on day 8 when the innate response and corresponding organ dysfunction are at a minimum (**Figure 5B**), although there are hints of association to survival on day 3 when we see the innate response begin to terminate (**Figure 5C**).

#### Metabolic

The metabolic module’s phenotypic (**Figure 4**) and temporal profile (**Figure 5B**) is indistinguishable from the innate module, and they share similar correlations in the eigengene network (Fig. 2B). In contrast to the innate module’s Cox profile, the metabolic module shows a consistent effect on survival outcomes across postoperative timepoints, with levels in non-survivors being elevated (**Figure 5C**). One difference in the make-up of these two modules is the type of immune cell markers that are observed within each module. An examination of the 100 genes with highest intramodular connectivity within each module reveals that the innate immunity module is enriched (*p*=.0016) for monocyte markers while the metabolic module is enriched (*p*=.011) for neutrophil markers. Neutrophils have previously been related to organ dysfunction [^12^](#_ENREF_12), and may explain the difference in Cox profile compared to the monocyte-rich innate immunity module.

### Metabolic supercluster

#### Demethylation

The demethylation module is seemingly unconnected to the rest of the eigengene network (**Figure 3B**). The module is filled with Y-chromosomal genes (Benjamini-Hochberg corrected *p*=3.3E-11) and X chromosomal genes (Benjamini-Hochberg corrected *p*=3.9E-4). Upon inspection of intramodular connectivity, we find that genes involved in X-inactivation (*Xist* and *Tsix*) are hubs of this module. However, we believe this module to be an artifact of our study design. WGCNA seeks to find groups of genes whose expression varies in a similar way across samples. Y-chromosomal and X-inactivation genes are only expressed in men and women, respectively. Therefore, clear expression patterns for these genes will be detected by WGCNA, and these genes will be grouped together into a single module. We find enrichment for H3K4 demethylases, likely due to the fact that there are very few of these demethylases, and one each is located on the Y and X chromosomes. In the Cox model, we find it to be consistently predictive of survival across all timepoints (**Figure 5C**), but given that all non-survivors in our study were male, we find it likely that this is the proper explanation for the association.

#### Coagulation

Clinical coagulation management to prevent bleeding (i.e. platelet transfusions) and clotting (i.e. anticoagulation medication), is considered important to the survival of patients with MOD ^[13](#_ENREF_13)^. The coagulation module shows a strong correlation with platelet count, both overall (Fig. 3) and temporally (Figs. 1B and 4B). This module shows strong enrichments for coagulation and platelet aggregation, and shows cell-type specificity for megakaryocytes. We further observe that several known repressors of clot degradation are present in this module as well. When the top 100 genes most significantly associated with the SOFA score are examined, we also observe enrichment for mitosis and cell division pathways, suggesting that the module's effects are likely due to platelet maturation.

#### Mitochondria

The mitochondria module is strongly associated with worsening organ dysfunction (**Figure 4**), and it is temporally synchronized with the innate immune response (**Figure 5C**). This has previously been noted in the literature [^31^](#_ENREF_31). Several platelet mitochondrial respiratory-chain enzymes are known to be inhibited during human sepsis and organ dysfunction, e.g. cardiogenic shock [^63^](#_ENREF_63), and mitochondrial biogenesis is associated with survival in MOD [^32^](#_ENREF_32)^,^ [^64^](#_ENREF_64). Pathway analysis revealed enrichment of the PAR/Thrombin pathway, which helps initiate coagulation. The activation of this pathway signals production of ROS in the mitochondria, and its activation after MCS surgery may contribute to adverse outcomes [^65^](#_ENREF_65). Pathway analysis revealed relationships to hub genes such as cytokines IFN-γ and IL-8, as well as the signal transduction gene MAPK1. These may indicate processes associated with macrophage activation, as well as recruitment of other innate immune cells (IFN-γ and MAPK1 activate macrophages, while IL-8 stimulates trafficking of other cells to site of infection). Another pathway hub identified with the mitochondria module is BLC2, which has a role regulating apoptosis [^33^](#_ENREF_33).

#### Ribosome

The ribosome module upregulates immediately following surgery, and remains activated throughout recovery (**Figure 5B**). As the body recovers from surgery, robust protein translation is an important component of many vital cellular processes, including cell division and the response to external stimuli and stressors. Furthermore, ribosomal proteins may play a protective role in host immune response by boosting immune signaling [^66^](#_ENREF_66). In the Cox profile (**Figure 5C**), we observe underexpression in non-survivors on day 1, followed by overexpression on day 3.

### Catabolic supercluster

#### Protein folding

The protein-folding module shares a similar time profile with the ribosome module (**Figure 5B**). It is strongly activated on day 8 along with the coagulation, catabolism, and apoptosis modules, but is also mildly upregulated on the day after surgery. Unlike the ER module, which appears to be enriched for stress-related chaperone proteins, this module is enriched for *de novo* posttranslational protein folding-associated genes. Additionally, there is a slight enrichment for genes involved in cytoskeleton maintenance and organization, suggesting that this module may be involved in the creation and maintenance of new cells after the catabolic processes have finished.

#### Catabolism

The catabolism eigengene is negatively associated with the SOFA score (**Figure 4**) and in many ways behaves similar to the coagulation module in terms of its relevance to MOD. Like the coagulation module, it is progressively activated throughout the study timecourse. It also appears to decrease mortality risk on day -1 and day 5 (**Figure 5C**). Because a relevant therapy for the prevention of MOD mortality is the use of anti-clotting agents such as warfarin after surgery has ended, our initial assumption was that this module would correspond to this function. To our surprise, none of the major members of the clot removal cascade are found in the module; there are very few ECM remodeling genes present. Instead, examination revealed a number of macrophage, autophagy and apoptosis-related genes, suggesting that the module is involved in the ubiquitination cascade and whole-cell turnover. We examined the top 100 genes in the module based on intramodular connectivity, and observe that these important genes are enriched for apoptosis and the adaptive immune response, as well as enrichment for genes expressed in proerythrocytes and genes that are causal for premature red cell death and anemia. Put together, these data suggest that this module is involved in the maintenance of blood homeostasis, ensuring that a proper amount of erythrocytes are produced in the body and acting to eliminate excess RBCs and WBCs that are produced. This matches well with the final predictive model, which included several genes involved in blood homeostasis and cell division.

#### RNA processing

The RNA processing eigengene is correlated with the catabolism eigengene (**Figure 3B**). RNA processing is an important component of the cell growth, division and response to external stressors. Its activation on the final two days suggests that as the body recovers from surgery, the reparative processes are being robustly activated (**Figure 5B**).

### Reparative supercluster

#### ER (Endoplasmic Reticulum)

The ER eigengene rises continually after surgery (**Figure 5B**) and shows a strong enrichment for the ER unfolded protein response, suggesting that this module is involved in the management of the intracellular reparative response after surgery.

#### Mitosis

The mitosis module is strongly enriched for genes involved in cell cycle maintenance and mitosis in general, and shows enrichment for markers of proerythrocytes, the precursor cell of erythrocytes. This is notable because of the enrichment seen in the catabolism module for erythrocyte cell markers, and the genes in the predictive model that correspond to blood homeostasis. The mitosis module is correlated with the catabolism module (**Figure 3B**), and peaks at day 5 (**Figure 5B**). The Cox model identifies it as associated with survival on days 5 and 8 (**Figure 5C**). The late-stage activation of this module suggests that mitosis of erythrocytes, as well as platelets (as in the coagulation module), are important to repair and survival with MOD.

#### Defense

The defense module shares several features with the mitosis, coagulation and catabolism modules. Like these modules, it is progressively activated over the course of the study (**Figure 5B**), and like the mitosis module, it is predictive of survival late in the timecourse (**Figure 5C**). It shows a significant association with worsening bilirubin (**Figure 4**), and enriched for genes involved in the innate immune system, specifically granulocytes and neutrophils. Neutrophil dysregulation has previously been associated with negative outcomes in MOD [^12^](#_ENREF_12). More broadly, granulocytes act to regulate inflammation in the body. Additionally, this module shows enrichment for a number of ECM remodeling proteins, suggesting that the granulocytes are assisting in the degradation of platelet clots (whose degradation is suggested by the beneficial role of anticoagulants in treatment of MOD), the freeing of dying cells from the ECM, or negatively affecting the integrity of the endothelial cell wall.

### Adaptive immune supercluster

#### B cells

The activity of the B cell module follows an interesting timecourse: it peaks on day 3 after surgery, in an intermediate phase between the innate immune response and the adaptive immune response (**Figure 5B**). The Cox model shows a very slight, but consistent, effect on survival across all timepoints (**Figure 5C**). One possible explanation is that non-survivors experience upregulation of plasma B cell subsets which are characteristically known to create further inflammation. We also find greater upregulation of IL-12B receptors in the non-surviving patients, across all timepoints. IL-12 is a critical regulator of the cell mediated Th1 response, and therefore is an important promoter of autoimmunity and inflammation. Finally, non-survivors may fail to express enough anti-inflammatory IL-10 immediately after surgery. IL-10, which is secreted by regulatory B cells (Bregs), plays a tremendous role in regulating the adaptive immune system and the inflammatory responses [^67^](#_ENREF_67). Immediately following surgery, we found that Il-10 is more highly expressed in survivors, suggesting that immediate Breg activity may be essential to survival post-surgery. Depending on the complex interplay between the B cell subsets, the inflammation response can hasten or subside, thereby drastically affecting recovery after MCS surgery.

Many genes in this module are associated with B cell activation and proliferation, such as CD19, MS4A1 (CD20), and CD22. CD20 and CD22 have been studied in context of inflammation and autoimmune diseases [^68^](#_ENREF_68). Furthermore, pathway analysis revealed enrichment in signal transduction through the B cell and Par 1 receptors. The high expression of genes in this module may indicate that the activation of B cells in particular are propagating the inflammation, and even assist in instigating coagulation processes via thrombin/Par1 pathway. However, another important factor in interpreting this module is the potential presence of Bregs, which mediate several anti-inflammatory events [^67^](#_ENREF_67).

#### Transcription

Like the B cell module, the transcription module peaks on day 3 (**Figure 5B**). This module is anti-correlated with the apoptosis, metabolic, innate, and mitochondria modules (**Figure 3B**). Since the innate, apoptosis and metabolic modules are associated with increased innate immune cell function, and the mitochondrial module is strongly associated with organ dysfunction (**Figure 4**), it seems likely that the transcription that is occurring in this module is linked to the role of the adaptive immune system in regulating the response of the body after the initial inflammatory phase is completed. Robust gene expression changes are important for proper responses to stressors and external stimuli, and the anti-correlation of this module to the four modules listed above suggests that the genes being transcribed are acting beneficially.

#### T cells

The T cell module is the largest module in the adaptive immune supercluster (**Figure 3A**), and is the module most strongly associated with improvement of organ dysfunction (**Figure 4**). Its expression is highly suppressed during the initial innate immune response, and then it steadily increases over the timecourse (**Figure 3B**), exhibiting a statistically significant association with the platelet count (**Figure 4**). The Cox model shows a mild negative association with survival, along with the NK cells and B cells, contrary to the association with improving organ failure scores (**Figure 3C**). Although this association with survival did not achieve statistical significance, it is fairly consistent across timepoints, suggest the effect is real. This is not necessarily paradoxical, as high levels of T cells are the result of the body’s attempt to deal with advanced disease. There may be a simple analogy to explain this result: firefighters put out fires, but the presence of a large number of firefighters at a given fire is indicative of a fire too large to control. T cells have previously been reported to play an important role in the regulation of organ failure, with the depletion of peripheral blood CD4+ T-cells being associated with persistent organ failure [^69^](#_ENREF_69). T cells have also been consistently shown to play an important role in inflammation-based organ damage ^[70](#_ENREF_70)^. Furthermore, these cells play a vital role in transplant rejection by mediating T-cell induced rejection ^[71](#_ENREF_71)^. Taken together, the proper regulation of this module acts to suppress organ dysfunction.

#### NK cells

The NK cell module has a similar profile to the T cell module. Like T cells, it is highly suppressed during the innate immune response, and continually rises throughout the rest of the timecourse (**Figure 5B**). It also shares a similar Cox profile with the T cell module (**Figure 5C**). NK cells play an important role in assisting T cells in host defense [^72^](#_ENREF_72), and when improperly regulated, may result in widespread organ failure [^73^](#_ENREF_73) and auto-immune disorders [^74^](#_ENREF_74). It likely acts in concert with the T-cell module to modulate the immune response to the surgery and, when properly controlled, protects the host from multi-organ dysfunction.

#### Type I IFN (Interferon)

The type I IFN module contains pathways and genes related to signal transduction—particularly with interferon, leukocyte proliferation, and leukocyte trafficking in both innate and adaptive processes. It shares pathways with the innate immune system, but is temporally anti-correlated with the innate response (**Figure 5B**). The module is heavily downregulated after surgery, and then increases expression at the later timepoints. This module becomes predictive of survival on the final day 8 (**Figure 5C**), confirming the previously described potential of type-I IFN to restore immunocompetence and improve survival outcomes [^75^](#_ENREF_75). Considering this is a later stage in the time course, and that genes in this module tend to be enriched (*p*=.011) for helper T-cell markers, this module may be associated with T cell function.

# Tables Supplement

## Modified SOFA score

**Table S1: Modified SOFA score.**

| **Score** | **0** | **1** | **2** | **3** | **4** |
| --- | --- | --- | --- | --- | --- |
| **Respiration** |  |  |  |  |  |
| Respiratory Status^*^ | -- | No respiratory support | -- | On mechanical ventilator | -- |
| **Coagulation** |  |  |  |  |  |
| Platelets (x1000/uL)^†^ | > 150 | < 150 | < 100 | < 50 | < 25 |
| **Liver** |  |  |  |  |  |
| Bilirubin (mg/dL) | < 1.2 | 1.2 - 1.9 | 2.0 - 5.9 | 6.0 - 11.9 | > 12.0 |
| **Cardiovascular** |  |  |  |  |  |
| Mean arterial pressure (mmHg)^‡^ | No hypotension | MAP<70 mmHg with no drugs | MAP<70 mmHg, one pressor medication | MAP<70 mmHg, one or more pressor medication at moderate dose | MAP<70 mmHg, one or more pressor medication at high dose |
| **CNS** |  |  |  |  |  |
| Glasgow Coma Scale^¶^ | 15 | 13 - 14 | 10 - 12 | 6 - 9 | < 6 |
| **Renal** |  |  |  |  |  |
| Creatinine | < 1.2 | 1.2 - 1.9 | 2.0 - 3.4 | 3.5 - 4.9 | > 5.0 |

* (Modified) PaO2 and FiO2 values are not recorded on medical charts for PaO2/FiO2 ratio calculation

† Corrected for platelet transfusion

‡ (Modified) Pressor medication given to patients are presented in several different metric units. Therefore, the CV score was modified into a general categorization of medication dosage level.

¶ Neurologic status calculated using the Glasgow Coma Scale (Teasdale *et al*). Sedated patients are given a GCS score of 3 (CNS SOFA score of 4) based on the Ramsay Sedation Scale (Ramsay *et al*).

## Gene Onotology Enrichment Analysis

**Table S2: Gene Onotology Enrichment Analysis.** We used GoSIM to infer gene ontology enrichments. For each module, we show the top 10 enriched terms, in each of the 3 categories of biological processes, molecular functions, and cellular components.

|  | **Module** | **Biological processes** | **Molecular functions** | **Cellular components** |
| --- | --- | --- | --- | --- |
|  | **Innate** |  |  |  |
|  |  | regulation of innate immune response | phospholipid binding | plasma membrane |
|  |  | activation of innate immune response | phosphatidylinositol binding | membrane |
|  |  | cellular response to bacterial lipopepti | lipid binding | cell periphery |
|  |  | detection of molecule of bacterial origi | signaling pattern recognition receptor a | intrinsic component of membrane |
|  |  | cellular response to stimulus | GTPase regulator activity | integral component of membrane |
|  |  | positive regulation of innate immune res | Notch binding | plasma membrane part |
|  |  | innate immune response-activating signal | solute:hydrogen antiporter activity | integral component of plasma membrane |
|  |  | response to stimulus | signaling receptor activity | intrinsic component of plasma membrane |
|  |  | phosphatidylglycerol biosynthetic proces | cell adhesion molecule binding | lysosome |
|  |  | Notch signaling pathway | GTPase activator activity | vacuole |
|  | **Apoptosis** |  |  |  |
|  |  | signaling | cytokine receptor binding | I-kappaB/NF-kappaB complex |
|  |  | cell communication | cytokine activity | Bcl3/NF-kappaB2 complex |
|  |  | regulation of cellular process | chemokine activity | extracellular space |
|  |  | response to molecule of bacterial origin | sequence-specific DNA binding | external side of plasma membrane |
|  |  | regulation of apoptotic process | chemokine receptor binding | CD40 receptor complex |
|  |  | response to lipopolysaccharide | MAP kinase tyrosine/serine/threonine pho | nucleoplasm |
|  |  | regulation of cell death | MAP kinase phosphatase activity | receptor complex |
|  |  | signal transduction | protein binding | voltage-gated sodium channel complex |
|  |  | regulation of programmed cell death | molecular_function | T-tubule |
|  |  | response to external stimulus | RNA polymerase II activating transcripti | annulate lamellae |
|  | **Metabolic** |  |  |  |
|  |  | endosomal transport | GDP binding | early endosome |
|  |  | regulation of intracellular signal trans | Rab guanyl-nucleotide exchange factor ac | intracellular membrane-bounded organelle |
|  |  | GTP metabolic process | SH3 domain binding | cyclin-dependent protein kinase holoenzy |
|  |  | entrainment of circadian clock | GTP binding | perinuclear region of cytoplasm |
|  |  | cleavage furrow formation | nucleic acid binding transcription facto | extrinsic component of membrane |
|  |  | epithelial cell differentiation involved | sequence-specific DNA binding transcript | endomembrane system |
|  |  | phosphate-containing compound metabolic | phosphatidic acid binding | recycling endosome membrane |
|  |  | GTP catabolic process | SNAP receptor activity | vesicle |
|  |  | regulation of phosphorus metabolic proce | RNA polymerase II regulatory region sequ | extracellular vesicular exosome |
|  |  | platelet-derived growth factor receptor | guanyl ribonucleotide binding | endosome |
|  | **Demethylation** |  |  |  |
|  |  | histone H3-K4 demethylation | histone demethylase activity | stereocilia ankle link complex |
|  |  | negative regulation of dendritic cell di | demethylase activity | viral envelope |
|  |  | regulation of dendritic cell differentia | dioxygenase activity | guanyl-nucleotide exchange factor comple |
|  |  | histone lysine demethylation | histone demethylase activity (H3-K4 spec | Gemini of coiled bodies |
|  |  | histone demethylation | oxidoreductase activity, acting on paire | SMN complex |
|  |  | protein demethylation | RNA polymerase II core promoter proximal | polysome |
|  |  | spliceosomal complex assembly | core promoter proximal region sequence-s | SMN-Sm protein complex |
|  |  | positive regulation of circadian sleep/w | bent DNA binding | ribonucleoprotein complex |
|  |  | positive regulation of circadian sleep/w | core promoter proximal region DNA bindin | preribosome |
|  |  | dendritic cell differentiation | translation initiation factor activity | U12-type spliceosomal complex |
|  | **Coagulation** |  |  |  |
|  |  | wound healing | actin binding | platelet alpha granule |
|  |  | blood coagulation | receptor binding | platelet alpha granule lumen |
|  |  | hemostasis | protein complex binding | cell periphery |
|  |  | regulation of body fluid levels | cytoskeletal protein binding | cell junction |
|  |  | platelet activation | G-protein coupled receptor binding | adherens junction |
|  |  | response to wounding | GTPase activity | secretory granule lumen |
|  |  | platelet degranulation | collagen binding | secretory granule |
|  |  | exocytosis | integrin binding | plasma membrane |
|  |  | cellular component movement | guanyl ribonucleotide binding | cytoplasmic membrane-bounded vesicle lum |
|  |  | cell motility | protein heterodimerization activity | cell projection |
|  | **Unclustered** |  |  |  |
|  |  | cerebellum morphogenesis | peptidyl-prolyl cis-trans isomerase acti | granular component |
|  |  | synaptic transmission | dopamine binding | myelin sheath |
|  |  | hindbrain morphogenesis | isomerase activity | cell body |
|  |  | metencephalon development | acetylcholine-activated cation-selective | lateral plasma membrane |
|  |  | neurological system process | bisphosphoglycerate mutase activity | presynaptic active zone |
|  |  | regulation of gene silencing by miRNA | bisphosphoglycerate 2-phosphatase activi | I band |
|  |  | cell-cell signaling | phosphoglycerate mutase activity | transcription elongation factor complex |
|  |  | cerebellar cortex morphogenesis | calcium-transporting ATPase activity | acetylcholine-gated channel complex |
|  |  | cerebellar cortex development | protein-glutamine gamma-glutamyltransfer | sarcoplasmic reticulum |
|  |  | inner ear morphogenesis | endoribonuclease activity, producing 5'- | perichromatin fibrils |
|  | **Mitochondria** | |  |  |
|  |  | respiratory electron transport chain | NADH dehydrogenase activity | respiratory chain |
|  |  | electron transport chain | NADH dehydrogenase (ubiquinone) activity | mitochondrial respiratory chain |
|  |  | cellular respiration | oxidoreductase activity, acting on NAD(P | mitochondrial inner membrane |
|  |  | mitochondrial ATP synthesis coupled elec | oxidoreductase activity, acting on NAD(P | organelle inner membrane |
|  |  | oxidation-reduction process | hydrogen ion transmembrane transporter a | mitochondrial respiratory chain complex |
|  |  | oxidative phosphorylation | oxidoreductase activity | organelle membrane |
|  |  | energy derivation by oxidation of organi | CTD phosphatase activity | mitochondrial membrane |
|  |  | generation of precursor metabolites and | electron carrier activity | mitochondrial envelope |
|  |  | mitochondrial electron transport, NADH t | proton-transporting ATP synthase activit | mitochondrion |
|  |  | proton transport | ubiquinol-cytochrome-c reductase activit | extracellular vesicular exosome |
|  | **Ribosome** | |  |  |
|  |  | translational elongation | structural constituent of ribosome | ribosome |
|  |  | translational termination | RNA binding | cytosolic ribosome |
|  |  | SRP-dependent cotranslational protein ta | poly(A) RNA binding | ribonucleoprotein complex |
|  |  | protein targeting to ER | structural molecule activity | cytosolic large ribosomal subunit |
|  |  | cotranslational protein targeting to mem | nucleic acid binding | large ribosomal subunit |
|  |  | establishment of protein localization to | rRNA binding | small ribosomal subunit |
|  |  | nuclear-transcribed mRNA catabolic proce | organic cyclic compound binding | cytosolic small ribosomal subunit |
|  |  | protein localization to endoplasmic reti | ribonucleoprotein complex binding | macromolecular complex |
|  |  | translation | mRNA binding | nuclear lumen |
|  |  | viral transcription | pyrimidine nucleotide binding | cytoplasm |
|  | **Protein Folding** |  |  |  |
|  |  | 'de novo' posttranslational protein fold | unfolded protein binding | zona pellucida receptor complex |
|  |  | 'de novo' protein folding | poly(A) RNA binding | chaperonin-containing T-complex |
|  |  | protein folding | RNA binding | prefoldin complex |
|  |  | RNA processing | phosphatidylinositol 3-kinase binding | caveola |
|  |  | STAT protein import into nucleus | mRNA binding | microtubule |
|  |  | positive regulation of phosphoprotein ph | dihydrofolate reductase activity | paraspeckles |
|  |  | positive regulation of tyrosine phosphor | histone kinase activity (H3-Y41 specific | T-tubule |
|  |  | central nervous system neuron differenti | SH2 domain binding | nuclear matrix |
|  |  | tyrosine phosphorylation of Stat5 protei | [acyl-carrier-protein] S-malonyltransfer | nuclear periphery |
|  |  | pteridine-containing compound biosynthet | phenylalanine 4-monooxygenase activity | Prp19 complex |
|  | **Catabolism** | |  |  |
|  |  | cellular protein catabolic process | ligase activity | hemoglobin complex |
|  |  | protein ubiquitination | acid-amino acid ligase activity | intracellular |
|  |  | ubiquitin-dependent protein catabolic pr | small conjugating protein ligase activit | cytoplasm |
|  |  | proteolysis involved in cellular protein | ubiquitin-protein ligase activity | cytosol |
|  |  | modification-dependent protein catabolic | oxygen transporter activity | intracellular membrane-bounded organelle |
|  |  | protein modification by small protein co | oxygen binding | ubiquitin ligase complex |
|  |  | protein polyubiquitination | catalytic activity | Cul4-RING ubiquitin ligase complex |
|  |  | autophagy | neutral amino acid transmembrane transpo | autophagic vacuole |
|  |  | protein catabolic process | enzyme regulator activity | intracellular organelle |
|  |  | protoporphyrinogen IX metabolic process | ammonium transmembrane transporter activ | protein complex |
|  | **RNA processing** | |  |  |
|  |  | monoubiquitinated protein deubiquitinati | calcium-induced calcium release activity | mRNA cap binding complex |
|  |  | embryonic heart tube development | P-P-bond-hydrolysis-driven protein trans | mitochondrial inner membrane presequence |
|  |  | regulation of heart rate by chemical sig | hydrolase activity, acting on ester bond | activin responsive factor complex |
|  |  | regulation of cellular respiration | intracellular ligand-gated calcium chann | PR-DUB complex |
|  |  | chondroitin sulfate catabolic process | protein transmembrane transporter activi | cone cell pedicle |
|  |  | RNA processing | L-iduronidase activity | basal cortex |
|  |  | regulation of intracellular steroid horm | UDP-glucose 6-dehydrogenase activity | cullin-RING ubiquitin ligase complex |
|  |  | positive regulation of calcium ion trans | iduronate-2-sulfatase activity | condensed nuclear chromosome, centromeri |
|  |  | glycosylation | glycoprotein endo-alpha-1,2-mannosidase | intraciliary transport particle |
|  |  | determination of left/right symmetry | pyridoxamine-phosphate oxidase activity | SCF ubiquitin ligase complex |
|  | **ER** | |  |  |
|  |  | endoplasmic reticulum unfolded protein r | dolichyl-diphosphooligosaccharide-protei | endoplasmic reticulum |
|  |  | cellular response to unfolded protein | oligosaccharyl transferase activity | endoplasmic reticulum lumen |
|  |  | response to endoplasmic reticulum stress | protein disulfide oxidoreductase activit | endoplasmic reticulum membrane |
|  |  | ER-nucleus signaling pathway | protein disulfide isomerase activity | nuclear outer membrane-endoplasmic retic |
|  |  | response to unfolded protein | disulfide oxidoreductase activity | endomembrane system |
|  |  | cellular response to topologically incor | oxidoreductase activity, acting on a sul | oligosaccharyltransferase complex |
|  |  | cell redox homeostasis | intramolecular oxidoreductase activity | melanosome |
|  |  | glycerol ether metabolic process | unfolded protein binding | integral component of endoplasmic reticu |
|  |  | protein N-linked glycosylation via aspar | isomerase activity | endocytic vesicle lumen |
|  |  | activation of signaling protein activity | ribonucleoprotein complex binding | intrinsic component of endoplasmic retic |
|  | **Mitosis** |  |  |  |
|  |  | mitotic cell cycle | ATP binding | chromosome |
|  |  | cell cycle | adenyl nucleotide binding | spindle |
|  |  | cell cycle process | purine ribonucleoside triphosphate bindi | microtubule cytoskeleton |
|  |  | cell division | protein binding | condensed chromosome |
|  |  | mitosis | purine nucleoside binding | protein-DNA complex |
|  |  | chromosome segregation | ribonucleoside binding | chromosome, centromeric region |
|  |  | DNA metabolic process | nucleoside binding | nuclear lumen |
|  |  | mitotic cell cycle phase transition | purine nucleotide binding | nucleus |
|  |  | cell cycle phase transition | tubulin binding | condensed chromosome, centromeric region |
|  |  | organelle organization | microtubule binding | cytoskeleton |
|  | **Defense** |  |  |  |
|  |  | defense response to bacterium | glycosaminoglycan binding | specific granule |
|  |  | response to bacterium | serine-type endopeptidase activity | secretory granule |
|  |  | defense response to fungus | leukotriene-B4 20-monooxygenase activity | azurophil granule |
|  |  | response to fungus | alpha-tocopherol omega-hydroxylase activ | extracellular region |
|  |  | killing of cells of other organism | low-density lipoprotein receptor activit | extracellular space |
|  |  | response to other organism | heparin binding | anchored component of membrane |
|  |  | antibacterial humoral response | serine-type peptidase activity | extracellular matrix |
|  |  | response to biotic stimulus | endopeptidase activity | lateral plasma membrane |
|  |  | growth of symbiont in host | serine hydrolase activity | extracellular vesicular exosome |
|  |  | killing by host of symbiont cells | chitin binding | membrane-bounded vesicle |
|  | **B Cells** |  |  |  |
|  |  | B cell activation | protein binding, bridging | external side of plasma membrane |
|  |  | humoral immune response | SH3/SH2 adaptor activity | integral component of plasma membrane |
|  |  | B cell proliferation | binding, bridging | intrinsic component of plasma membrane |
|  |  | B cell differentiation | signaling adaptor activity | cell periphery |
|  |  | B cell receptor signaling pathway | guanyl-nucleotide exchange factor activi | integral component of membrane |
|  |  | leukocyte activation | extracellular matrix structural constitu | cell surface |
|  |  | lymphocyte activation | Ras guanyl-nucleotide exchange factor ac | intrinsic component of membrane |
|  |  | lymphocyte proliferation | opioid peptide activity | plasma membrane |
|  |  | mononuclear cell proliferation | cannabinoid receptor activity | plasma membrane part |
|  |  | leukocyte proliferation | MRF binding | membrane |
|  | **Transcription** |  |  |  |
|  |  | RNA metabolic process | DNA binding | nucleus |
|  |  | transcription, DNA-templated | nucleic acid binding | intracellular membrane-bounded organelle |
|  |  | regulation of transcription, DNA-templat | metal ion binding | Golgi transport complex |
|  |  | regulation of RNA biosynthetic process | cation binding | intracellular organelle |
|  |  | regulation of RNA metabolic process | nucleic acid binding transcription facto | nucleolus |
|  |  | regulation of gene expression | sequence-specific DNA binding transcript | holo TFIIH complex |
|  |  | cellular macromolecule biosynthetic proc | organic cyclic compound binding | Elongator holoenzyme complex |
|  |  | nucleobase-containing compound metabolic | taste receptor activity | axoneme |
|  |  | heterocycle metabolic process | pseudouridine synthase activity | core TFIIH complex |
|  |  | regulation of nitrogen compound metaboli | intramolecular transferase activity | euchromatin |
|  | **T Cells** |  |  |  |
|  |  | lymphocyte differentiation | receptor activity | plasma membrane |
|  |  | T cell differentiation | signaling receptor activity | cell periphery |
|  |  | homophilic cell adhesion | metal ion binding | integral component of membrane |
|  |  | T cell differentiation in thymus | cation binding | intrinsic component of membrane |
|  |  | T cell activation | transmembrane signaling receptor activit | plasma membrane part |
|  |  | cell-cell adhesion | signal transducer activity | synapse |
|  |  | alpha-beta T cell differentiation | Rho guanyl-nucleotide exchange factor ac | T cell receptor complex |
|  |  | cell adhesion | olfactory receptor activity | synaptic membrane |
|  |  | T cell selection | steroid hormone receptor activity | membrane |
|  |  | alpha-beta T cell activation | protein tyrosine kinase activity | proteinaceous extracellular matrix |
|  | **NK Cells** |  |  |  |
|  |  | cellular defense response | transmembrane signaling receptor activit | plasma membrane |
|  |  | immune response | receptor activity | cell periphery |
|  |  | regulation of immune response | signal transducer activity | plasma membrane part |
|  |  | immune system process | signaling receptor activity | external side of plasma membrane |
|  |  | signal transduction | MHC class I receptor activity | intrinsic component of plasma membrane |
|  |  | regulation of immune system process | G-protein coupled receptor activity | intrinsic component of membrane |
|  |  | defense response | prostaglandin J receptor activity | membrane |
|  |  | positive regulation of natural killer cells | prostaglandin-D synthase activity | integral component of plasma membrane |
|  |  | cell surface receptor signaling pathway | prostaglandin D receptor activity | integral component of membrane |
|  |  | signaling | coreceptor activity | cell surface |
|  | **Type I IFN** |  |  |  |
|  |  | defense response | MHC class II receptor activity | MHC class II protein complex |
|  |  | immune response | double-stranded RNA binding | integral component of lumenal side of en |
|  |  | cytokine-mediated signaling pathway | single-stranded RNA binding | ER to Golgi transport vesicle membrane |
|  |  | innate immune response | peptide antigen binding | ER to Golgi transport vesicle |
|  |  | cellular response to cytokine stimulus | helicase activity | clathrin-coated endocytic vesicle membra |
|  |  | response to cytokine | antigen binding | trans-Golgi network membrane |
|  |  | defense response to virus | nucleoside-triphosphatase activity | clathrin-coated endocytic vesicle |
|  |  | immune system process | threonine-type endopeptidase activity | transport vesicle membrane |
|  |  | type I interferon signaling pathway | 2'-5'-oligoadenylate synthetase activity | integral component of endoplasmic reticu |
|  |  | cellular response to type I interferon | pyrophosphatase activity | intrinsic component of endoplasmic retic |

## Pathway analysis and transcription factor binding site enrichments

**Table S3:** **Pathway analysis and transcription factor binding site enrichments.** We conducted pathway analyses using Strand NGS bioinformatics software. We identified the top pathways and their hubs for each module. In addition, we used Whole-Genome rVista to analyze enrichment transcription factor binding sites in each module.

|  | **Module Name** | **Pathway Hubs (NLP)** | **Enriched Pathways** | **TF Binding Sites** |
| --- | --- | --- | --- | --- |
|  | **Innate** | TNF,INS,IL-1B,MAPK1,FOS,JUN ,NFKB1,IL-1A,IL4,CD40,CD40L,TLR4,TGFA,IL15,IL-22,TLR9,TLR3,TLR5,TLR1,IL6R,IL10RA,IL10RB,Il12RB2,CSFR2A | Signal Transduction, Innate Immune System, Regulation of RAC1 Activity, P53 Effectors, Signaling by GPCR, RAC1 signaling pathway | Zfp206,ERF,TFII-I,E2F-4 |
|  | **Apoptosis** | TNF,IL6,IL1B,HOOH,MAPK1,IL2,IL8,IL4,FOS,JUN,IL10,NFKβ | Signal Transduction, Innate Immune System, IL1-mediated signaling events, Direct p53 effectors, IL23-mediated signaling events | NF-kappaB, ATF, CREM, E4F1 |
|  | **Metabolic** | CDKN1A,NR3C1,CD44,IL18,ABCA1,IL16,CD55,IFNGR2 | Signal Transduction,N-cadherin signaling events, Posttranslational regulation of adhrens junction stability and dissassembly, Innate Immune System, P53 effectors, C-MYC pathway | E2F-4,Zfp206,ATF,XBP-1 |
|  | **Demethylation** | USP9Y,SERPIND1,UTS2,TNFRSF9,ADAMTS4 | GCPR ligand binding, Class A/1 Rhodopsin Receptors, Transcription Targets of AP1 family Fra 1 and Fra 2, TCR signaling in Naïve CD8 Cells | Egr-1,TGIF,HNF6,HIF1 |
|  | **Coagulation** | MAPK1,TGFB1,AKT1,SRC,AR,IL8RB | Signal Transduction, Extracellular Matrix Organization, Signaling by GCPR, Cell-cell communication, Proteoglycan syndecan mediated signaling events, Hemostasis, Cell Surface Interactions at the Vascular Wall, Integrins in Angiogenisis | SP2, SP4, EKLF,EGR-1 |
|  | **Unclustered** | MAPT,TF,HIST1H4I,RET,CBX8 | Signal Transduction, GPCR ligand binding, Signaling by GPCR, Class A/1 (Rhodopsin-like receptors), p63 transcription factor network | Staf, P50:P50, NF-muE1 |
|  | **Mitochondria** | MAPK1,IL8,IFNG,BCL2,MAPK3,IL10,STAT3,ICAM1,STAT1,IL5,IL8RA,IL8RB,IL23R,IL27RA,IL26 | Innate Immune System, Thrombin/protease activated PAR pathway, Par1-mediated thrombin signaling events, Regulation of RAC1 activity, C-Myc Pathway | SAP-1A,HFH3,C-ETS-1,Net |
|  | **Ribosome** | TNF, FASN,MAPK3,POMC,AHSA1,BAX | Innate Immune System, Signal Transduction, Proteoglycan syndecan mediated signaling events, p73 transcription factor network, C-MYC pathway, RAC1 signaling pathway, CXCR4-mediated signaling events | HFH3, SAP-1A,Net, SP2 |
|  | **Protein Folding** | IRS1,JAK2,HSPA8,ERBB4,PYY | Endothelins, Signaling Events mediated by PTP1B, C-MYB transcription factor network, Signal Transduction, ERBB4 signaling events | P50:P50,E2F-4,DP-1,Lhx3,NRF-1 |
|  | **Catabolism** | TNF,MAPK1,MYC,JUND,MAPK14 | Innate Immune System, C-MYC pathway, Factors involved in megakaryocyte development and platelet production, Factors involved in megakaryocyte development and platelet production, Regulation of RAC1 activity | SAP-1a, Net, Zfp206, E2F-4 |
|  | **RNA Processing** | TCEAL1,SKP2,IKBKG,SREBF1,FZR1,IL4R | Signal Transduction, Post translational Modification, Asparagine N linked glycosylation, Diabetes Pathways, FOXM1 Transcription Network, Aurora A Signaling | HFH3,SAP-1A,Irx2,HOXC13 |
|  | **ER** | IGF1,PPA1,CAV1,CYSLTR2,TP73 | Unfolded Protein Response, Direct p53 effectors, Activation of Chaperone Genes by XBP1(S), Activation of Chaperones by IRE1alpha, Activation of Chaperones by ATF6-alpha | HIF1, HIF-2alpha, AML1, ATF6 |
|  | **Mitosis** | BRCA1,E2F1,EPEG],CDC2,PCNA ,ATM | Cell Cycle, Signaling by Aurora kinases, E2F transcription factor network, Mitotic M-M/G1 phases, FOXM1 transcription factor network | E2F-4:DP1, E2F-4:DP-2, Rb:E2F-1:DP-1 |
|  | **Defense** | MMP9,LTF,MPO,MYB,ERG | Extracellular Matrix Organization, Degradation of Extracellular Matrix, CYM B transcription network, Collagen Degradation, Innate Immune System, Hemostasis | dlx3, K-2b,ALX-3,Gbx2 |
|  | **B Cells** | LYN,FCER2,CD19,MS4A1,CD22 | Thrombin/protease-activated receptor (PAR) pathway, PAR1-mediated thrombin signaling events, Class I, PI3K signaling events, BCR signaling pathway, Signaling events mediated by focal adhesion kinase | dlx3, Gbx2, FOXO4, Msx-1 |
|  | **Transcription** | D4S234E,PLAU,HLA-A,RUNX1,IL1R1 | Signal Transduction, Post-translational protein modification, , C-MYC pathway, Hemostasis, Biosynthesis of the N-glycan precursor (dolichol lipid-linked oligosaccharide, LLO) and transfer to a nascent protein, DNA Repair, Metabolism of lipids and lipoproteins | D4S234E, PLAU, HLA-A, RUNX1 |
|  | **T Cells** | TNF,TP53,IL1B,IL2,FOS,IL10,IL5,IL2RA | Signal Transduction, Developmental Biology, Signaling by GPCR, Axon guidance, GPCR ligand binding | Egr-1, Zfp206, SP1:SP3, ZBP89 |
|  | **NK Cells** | CLEC2D,OSBPL5,ERBB2,PHLDB2,GZMA,KLRG1 | Signal Transduction, TCR signaling in naïve CD8+ T cells, Immunoregulatory interactions between a Lymphoid and a non-Lymphoid cell, Immunoregulatory interactions between a Lymphoid and a non-Lymphoid cell, Signaling by GPCR | Egr-1, Zfp206, SP1:SP3, AML |
|  | **Type I IFN** | ICAM1,STAT1,PLAU,PML,ITGAL | Innate Immune System, Interferon gamma signaling, Signal Transduction, RIG-I/MDA5 mediated induction of IFN-alpha/beta pathways, Signal Transduction | ICSBP, ISGF-3 |

# Figures Supplement

## Module p-value distributions

**Figure S1: Module p-value distributions.** We assessed the p-distributions in each module in relationship to organ failure and survival. We marked the median p-values with a line, and used the binomial test around p=0.05 to assess the balance each p-distribution. Asterisks are shown for statistically significant modules (with Benjamini-Hochberg correction). **(A and B)** We computed p-values for each gene-phenotype association using a linear mixed effect model, and analyzed the p-distributions within each module. Note that almost all modules are enriched with genes associated with organ dysfunction, as measured by **(A)** SOFA and **(B)** MELD-XI scores, illustrating the systemic nature of the syndrome. The innate and adaptive immunity supercluster modules have the most skewed p-distributions. This result is consistent with the linear mixed model analysis of the corresponding eigengenes in Figure 3, which identifies strong correlations between organ dysfunction and the innate and adaptive immune eigengenes. Also note that the B Cell and Catabolism distributions are much more skewed for SOFA score than MELD-XI. **(C)** We computed p-values for each gene-survival association using a Cox model at each timepoint, and analyzed the p-distributions within each module. We note several changes in the p-distributions over time. The demethylation and T cell modules show skew across all timepoints, achieving significance multiple times. Prior to surgery, the protein-folding module has significant skew. Immediately following surgery, the RNA processing and ribosome modules become skewed. On day 3, we see skew in the apoptosis, innate, transcription, and T cell modules. On day 5 and day 8 days, the defense and mitosis modules both become highly skewed. Also on day 5, the catabolism and RNA processing modules become skewed, marking activation of both the reparative (yellow) and catabolic (green) superclusters. On day 8, we see significant skew in the type I IFN and transcription modules, as well as the innate and mitochondria, as the reparative (yellow) supercluster remains activated.
